# Photonic-circuited resonance fluorescence of single molecules with an ultrastable lifetime-limited transition

**DOI:** 10.1038/s41467-022-31603-x

**Published:** 2022-07-09

**Authors:** Penglong Ren, Shangming Wei, Weixi Liu, Shupei Lin, Zhaohua Tian, Tailin Huang, Jianwei Tang, Yaocheng Shi, Xue-Wen Chen

**Affiliations:** 1 School of Physics and Wuhan National Laboratory for Optoelectronics, Huazhong University of Science and Technology, Wuhan, People’s Republic of China; 2 Institute for Quantum Science and Engineering, Huazhong University of Science and Technology, Wuhan, People’s Republic of China; 3 Centre for Optical and Electromagnetic Research, State Key Laboratory for Modern Optical Instrumentation, College of Optical Science and Engineering, Zhejiang University, Zijingang Campus, Hangzhou, China

**Keywords:** Single photons and quantum effects, Quantum optics, Atomic and molecular interactions with photons, Quantum optics

## Abstract

Resonance fluorescence as the emission of a resonantly-excited two-level quantum system promises indistinguishable single photons and coherent high-fidelity quantum-state manipulation of the matter qubit, which underpin many quantum information processing protocols. Real applications of the protocols demand high degrees of scalability and stability of the experimental platform, and thus favor quantum systems integrated on one chip. However, the on-chip solution confronts several formidable challenges compromising the scalability prospect, such as the randomness, spectral wandering and scattering background of the integrated quantum systems near heterogeneous and nanofabricated material interfaces. Here we report an organic-inorganic hybrid integrated quantum photonic platform that circuits background-free resonance fluorescence of single molecules with an ultrastable lifetime-limited transition. Our platform allows a collective alignment of the dipole orientations of many isolated molecules with the photonic waveguide. We demonstrate on-chip generation, beam splitting and routing of resonance-fluorescence single photons with a signal-to-background ratio over 3000 in the waveguide at the weak excitation limit. Crucially, we show the photonic-circuited single molecules possess a lifetime-limited-linewidth transition and exhibit inhomogeneous spectral broadenings of only about 5% over hours’ measurements. These findings and the versatility of our platform pave the way for scalable quantum photonic networks.

## Abstract (web summary)

Single molecules can generate high-quality single photons for quantum technologies, but coupling to waveguides is difficult. Here, the authors show on-chip background-free resonance fluorescence generation and routing from single molecules with lifetime-limited transition and waveguide-aligned dipoles.

## Introduction

Resonantly driven single two-level quantum systems provide a powerful route to generate indistinguishable single photons on demand and to coherently control the internal state of the individual quantum systems, which are crucial for the implementations of various quantum information processing schemes^1–3^. On-chip integration of single quantum systems offers the appealing prospects of having high degrees of scalability and stability of the systems^4–7^, which are increasingly important for the developments towards real applications. In practice, hybrid integrated quantum photonic systems provide an attractive viable solution to combine high-performance solid-state quantum systems and advanced nanophotonic elements in large scale on one chip. Among various types of solid-state quantum systems^7,8^, quantum dots and color centers in diamond naturally have benefitted from the rapid development of nanofabrication technologies for inorganic semiconductor materials and become relatively more sophisticated in building on-chip quantum devices^5,9–12^. High-efficiency couplings of these quantum emitters with waveguides have been demonstrated by employing photonic nanostructures based on different operation principles^11,13–15^. Very recently, exciting progresses, including large-scale integration of color centers^16^ and low-background resonance fluorescence (RF) in waveguides from single quantum dot^14,17,18^, have been reported. However, despite impressive advances, the field of hybrid integrated quantum photonics still confronts several difficult challenges. For instance, the nanofabricated semiconductor structures readily result in charge fluctuations around the quantum systems and hence spectral wandering or diffusion of the emission energy from one photon to the other^6,11,19^, making the emitted photons indistinguishable only for a short-time delay. For quantum dots coupled to nanophotonic waveguides, the time delays for achieving indistinguishability are typically limited to tens of nanoseconds^14,17,18,20^. While lifetime-limited linewidths have been obtained with delicate control of the charge noise and fast scan^21,22^, spectral wandering persists and becomes a roadblock for scaling up the system size where longtime stable lifetime-limited transitions are required. Moreover, the randomness of the quantum emitters in spatial position, transition frequency and dipole orientation^6,20^ complicates the collective control of their interaction with the circuits.

Single molecules embedded in organic crystalline matrices are a class of bit less popular solid-state quantum systems^23^ but have been extensively studied in the context of biology, physical chemistry and spectroscopy^24^. Despite the usual impression that molecules suffer from photobleaching and broad spectra, it turns out that polycyclic aromatic hydrocarbons, such as dibenzoterrylene (DBT) embedded in anthracene (AC) or naphthalene crystal, can actually have narrow lines and be definitely photostable^25,26^. They have been demonstrated as stable single-photon emitters^26^, nearly ideal two-level quantum systems^27^, nanometer-sized acoustic detectors^28^ and flexible interfaces with alkali atoms^29^. Recently, single molecules also began to be actively explored for integrated quantum photonics^30^ due to their unique advantages of possessing stable lifetime-limited zero-phonon lines (ZPLs) at liquid helium temperature^24,25^ and of being small (~1 nm) suitable for doping at high densities^24^, which are both important for the scalability. Towards integrated molecular quantum devices, several groups have reported encouraging results on integrating single molecules onto a variety of nanophotonic circuit structures^26,31–36^. However, on-chip generation and routing of RF from single molecules with the suppression of excitation laser background have not been achieved. Moreover, the molecules near nanofabricated surfaces also tend to have inhomogeneous spectral fluctuations upon light excitation^33^. In addition, the existing integration schemes have difficulties to control the molecules’ dipole orientation, which is crucial for coupling with the nanophotonic elements. Therefore, a grand challenge in this line of research is to seamlessly integrate organic molecules to inorganic semiconductor nanostructures in a controlled and cryogenic-temperature compatible manner without introducing appreciable spectral wandering and scattering background^31,33^.

In this work we report an organic-inorganic hybrid quantum photonic platform that does meet the aforementioned key challenges and demonstrate photonic circuited background-free resonance fluorescence from a lifetime-limited-linewidth transition of single molecules with ultrahigh spectral stability. Here single DBT molecules embedded in an ultrathin AC crystal is hybridly integrated to silicon nitride (Si_3_N_4_) based waveguide circuits via a pick-and-place approach. The dipole orientations of the DBT molecules can be identified and collectively aligned with the waveguide structure. By applying spatial filtering in both real and Fourier spaces as well as polarization filtering, we could greatly suppress the same-frequency excitation laser background and demonstrate single-molecule resonance fluorescence in the waveguide with record-high signal to background ratios (SBRs) of over 3000. In addition, we show that the photonic-circuited molecules exhibit lifetime-limited ZPLs and possess such linewidth for hours over many excitation cycles without any feedback control. Our platform decouples the nanofabrication of the semiconductor photonic structures and the organic host materials, and thus allows the inclusion of various advanced nano-optical elements and microelectrodes to control the photonic coupling and the molecules, ensuring a high degree of scalability.

## Results

### Photonic-circuited single photons from single molecules

**Fig. 1:**
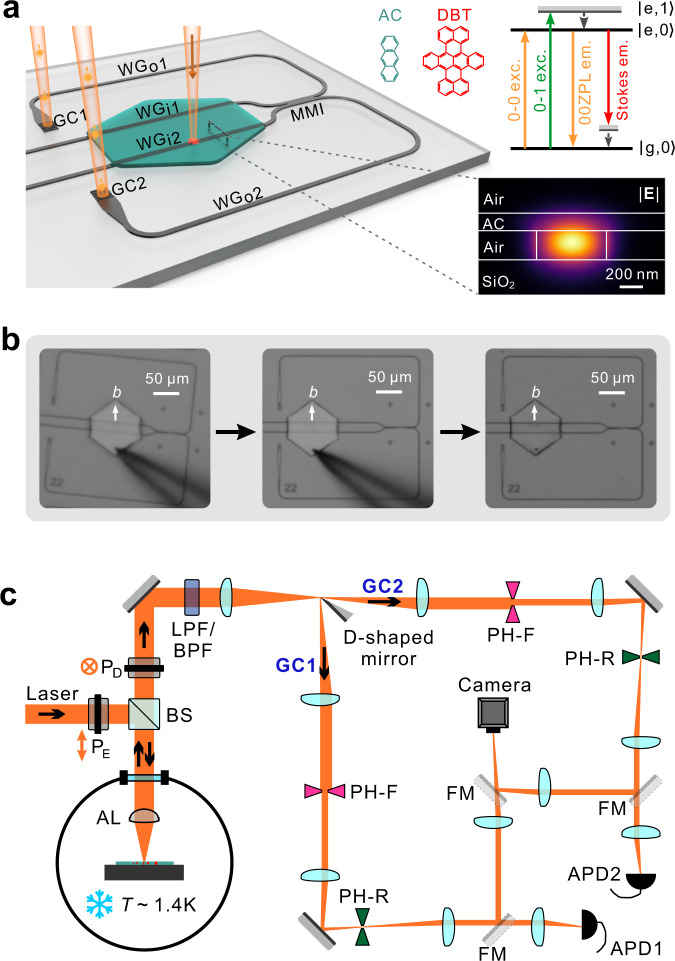
An organic-inorganic hybrid integrated quantum photonic platform and the experimental setup. **a** Schematic illustration of the platform consisting of a Si_3_N_4_-based photonic backbone and a crystalline anthracene (AC) nanosheet containing single dibenzoterrylene (DBT) molecules with dipole orientations aligned. The sample is excited from space and the emitted single photons are coupled to the waveguide, beam spilt through the MMI coupler and routed to the grating couplers (GC1 and GC2) for detection. Upper-right inset: Simplified Jabłoński diagram for relevant excitation and fluorescence processes. Lower-right inset: electric field distribution of the TE_0_ mode of the waveguide. **b** Optical micrographs showing the pick-and-place hybrid integration with the crystal orientation aligned with the waveguides. The white arrow denotes the “*b*” axis of the crystal. **c** Optical setup for the experiment (Supplementary Note 3). BS beamsplitter, AL aspheric lens, P_E/D_ polarizer for excitation/detection, FM flip mirror, PH-F pinhole in Fourier plane, PH-R pinhole in real image plane, LPF long-pass filter for Stokes-shifted fluorescence, BPF narrow band-pass filter for resonance fluorescence, APD avalanche photodiode single photon detector.

Figure 1a sketches the architecture and operation principle of the hybrid integrated quantum photonic system, which is comprised of a nanofabricated inorganic photonic structure, i.e., Si_3_N_4_ based waveguide circuits^37^, and an organic crystalline flake, i.e., an AC nanosheet with DBT molecules embedded^25,38^. The nanosheet is obtained through a co-sublimation process^25^ and has a hexagonal shape with an area of 7200 μm^2^ and a thickness of 150 nm (Supplementary Note 1)^39^. The thin thickness facilitates an evanescent coupling of DBT molecules to the fundamental transverse electric (TE_0_) mode of the waveguide (see the mode profile in the lower inset of Fig. 1a; Supplementary Note 2). Through a pick-and-place process illustrated in Fig. 1b, the nanosheet is integrated and bonded to the S_3_N_4_ waveguides via van der Waals forces. A crucial advantage of our system is that the dipole orientations of the embedded DBT molecules can be collectively aligned to the target waveguide during the assembly (Supplementary Note 1) since molecules’ dipole orientations are aligned to the “*b*” axis of the AC crystal^38^, recognizable from the hexagonal shape^40^. The AC nanosheet and the bonding remain robust in the helium bath cryostat with the temperature cooled down to 1.4 K. Under a narrow-band laser excitation from free space, individual molecules can be selectively excited and emit single photons into the waveguide, which are guided towards a 2 × 2 multi-mode interference (MMI) coupler for on-chip beam splitting^41^. The split streams of single photons are further routed by 560 μm before out-coupling to free space via two grating couplers (GC1 and GC2). According to the molecule levels indicated by the simplified Jabłoński diagram as in the top inset of Fig. 1a, a long-pass filter (LPF) is applied to collect the Stokes-shifted fluorescence while a narrow band-pass filter (BPF) is used for collecting RF, i.e., 00ZPL emission under resonant 0-0 excitation. As illustrated in Fig. 1c, the photon streams from GC1 and GC2 after passing the filter are separated by a D-shaped mirror and sent for further independent processing and detection (Supplementary Note 3).

**Fig. 2:**
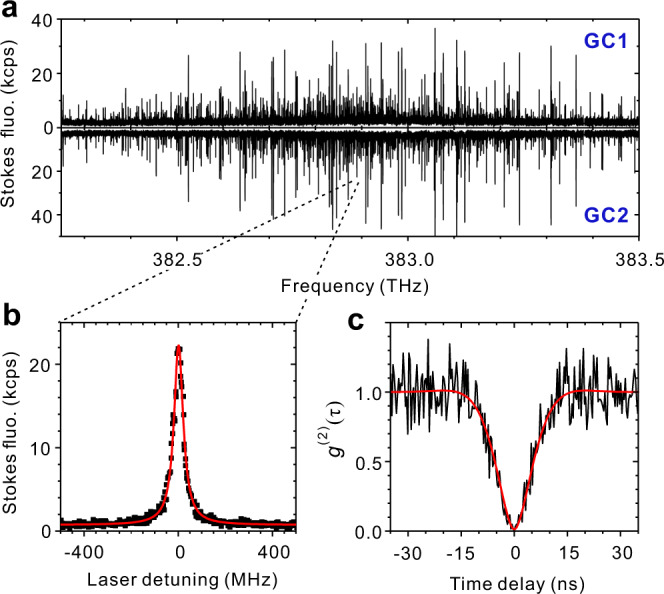
On-chip generation, beam-splitting and routing of single photons from densely-doped single DBT molecules. **a** Fluorescence-excitation spectra obtained by recording Stokes-shifted fluorescence from GC1 and GC2 as the excitation laser frequency is scanned across molecules’ 00ZPL inhomogeneous band of 1.25 THz. It is in wide-field illumination mode with a laser spot size of 15 μm. **b** High-resolution fluorescence-excitation spectrum of one single molecule. The laser frequency is scanned across the 00ZPL with a speed of 200 MHz/s. **c** Second-order cross-correlation function *g*^(2)^(τ) of Stokes-shifted fluorescence split on-chip to GC1 and GC2 from the same molecule in **b**. Here the excitation power is set to 11.21 nW (*S* = 0.75) and *g*^(2)^(0) = 0.013(1).

Figure 2a presents two fluorescence-excitation spectra obtained from recording the Stokes-shifted fluorescence from GC1 and GC2 as the laser frequency is scanned through molecules’ 00ZPL inhomogeneous band of 1.25 THz. Each spectrum include 710 narrow lines which correspond to this number of molecules situated in slightly different nanoscopic environment^24^. With a consideration of the laser spot size, we estimate every 20 nm length there is one molecule coupled to the waveguide. Such a high density of well-aligned quantum emitters in combination with microelectrodes for the Stark effect promises scalability of our platform^6^. Figure 2b displays a high-resolution excitation spectrum from one molecule. Second-order cross-correlation function *g*^(2)^(*τ*) of the Stokes-shifted fluorescence collected through GC1 and GC2 depicts an anti-bunching dip of *g*^(2)^(0) = 0.013(1) in Fig. 2c. The dip residue is mainly contributed by APD dark counts. Figure 2 has featured on-chip generation, beam splitting and routing of single photons from single DBT molecules.

### Photonic-circuited resonance fluorescence from single molecules

**Fig. 3:**
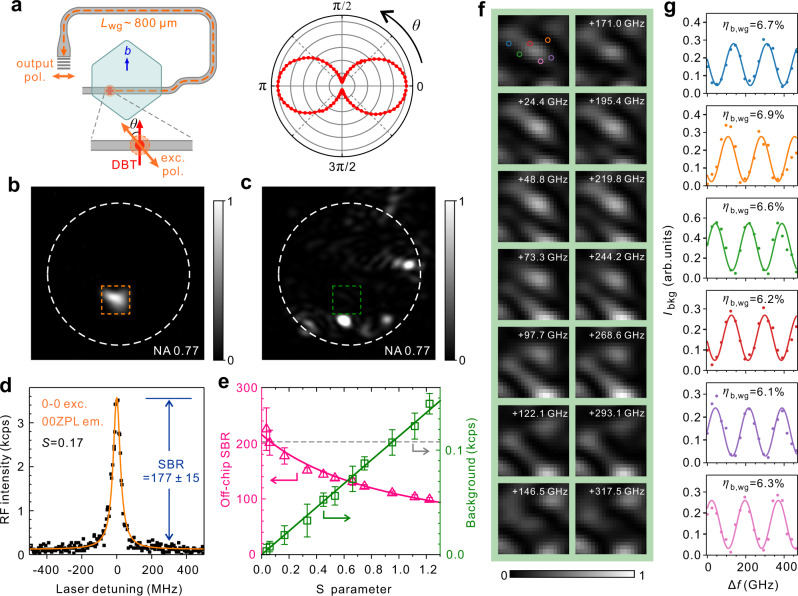
Photonic-circuited resonance fluorescence from a single molecule. **a** Left: schematic illustration of the crystal orientation, DBT molecule’s dipole orientation and the arrangement of the excitation and output-coupling polarizations; right: normalized output red-shifted fluorescence intensity as a function of the polarization of the excitation laser. **b** Fourier-plane image of the GC1-output 00ZPL emission of a single molecule under 0-1 excitation. The excitation laser is spectrally filtered. **c** Fourier-plane image of the laser background. Here the excitation is tuned 200 GHz away from the 00ZPL transition of the molecule and PH-R and polarization filtering have been applied. **d** RF-excitation spectrum of the molecule recorded through GC1 under excitation intensity *S* = 0.17. Here PH-F, PH-R and polarization filtering have been applied. **e** Pure background count rates without dark count (green, right y axis) and SBR (pink, left y axis) as functions of the saturation parameter *S*. Green squares represent the measured pure background count rates (error bars represent the standard deviations). Green solid line is a linear fit. Pink triangles represent the measured SBRs (error bars stem from the fitting errors in the measured RF count rates). Pink solid line displays the calculated SBR from the RF saturation curve (Supplementary Note 8). The gray dashed line denotes the dark count rate. **f** Background intensity pattern within the PH-F (see the dashed rectangle in **c**) as the change of the laser frequency (indicated at the upper-right corner of each pattern). Two columns cover two periods of pattern evolution. **g** Background intensity as a function of the laser frequency change. Each color-coded trace corresponds to a position marked in the first graph of **f**.

The above demonstration lays the groundwork for photonic-circuited RF with ultrahigh SBRs. The key to achieve this is to enhance the signal and suppress the same-frequency laser background. As shown in Fig. 3a, the alignment of molecule dipoles with the TE_0_ mode field asks for an aligned linearly polarized excitation to maximize the RF signal. This is faithfully confirmed by the polar plot of the emission intensity with the excitation polarization in the right panel of Fig. 3a. To suppress laser background, we take two innovative measures. Firstly, we exploit the advantage that the polarization state of the RF on the chip can be altered at will by bending the waveguide. Therefore we bend the photonic circuit in such a way that the output polarization is orthogonal to the excitation laser, enabling a cross-polarization detection to eliminate the excitation without blocking any signal. Secondly, we utilize the property that the output from the grating is directional and has a narrow distribution in the Fourier plane (Supplementary Note 2). Figure 3b shows a recorded Fourier-plane image (Supplementary Note 4) for the filtered 00ZPL fluorescence from GC1 for a molecule under 0-1 excitation. A small bright spot is observed. On the contrary, the laser background shows a broad speckle-like distribution in Fig. 3c. Therefore we place a pinhole at the Fourier plane, as indicated by the dashed rectangles in Fig. 3b, c, to block a large portion of background and allow the signal to pass. Besides applying the two above measures, a regular pinhole in the real-image plane is also used. These measures allow us to selectively suppress the laser background by over 7 orders of magnitude (Supplementary Note 5). Figure 3d displays a clean narrow RF-excitation spectrum recorded from GC1 under an excitation intensity level of *S* = 0.17, where *S* denotes the saturation parameter, i.e., the excitation intensity normalized by the saturation level (Supplementary Note 8). The excitation-intensity dependent laser background and SBR are presented in Fig. 3e, where the symbols are the measured data and the solid curves are the fittings. The SBR reaches 216 ± 10 at the weak excitation limit and decreases with the increase of the excitation intensity as expected, for instance, SBR = 108 ± 3 at *S* = 1.0.

Note that here the SBRs result from a particular off-chip detection scheme, where the sites of laser excitation and RF collection are quite close due to the constraint by the field of view of our optics. The scheme inevitably incorporates scattered laser background that is not waveguide-coupled. As a matter of fact, the directly scattered laser background dominates in the total background and leads to slightly different measured SBRs for the two grating couplers and for molecules from different positions (Supplementary Note 5). However, for applications in future developments, laser excitation and RF detection could be completely separated by using different approaches, for instance, routing RF to locations centimeters away, photonic wire bonding to optical fibers^42^ and on-chip integration of photon detectors^12^. Therefore, it is the waveguide-coupled laser background field **E**_b,wg_ that really matters and should be quantified. It turns out to be a highly nontrivial task to separate **E**_b,wg_ from the rest speckle-like scattered background field **E**_b,sp_ since they coherently interfere to form the final recorded intensity pattern ${\left|{{{{{{\bf{E}}}}}}}_{{{{{{\rm{b}}}}}},{{{{{\rm{wg}}}}}}}+{{{{{{\bf{E}}}}}}}_{{{{{{\rm{b}}}}}},{{{{{\rm{sp}}}}}}}\right|}^{2}$. Here we study the background intensity pattern as a function of the laser frequency and present the recorded images in Fig. 3f. The pattern at the central region exhibits a pronounced periodic evolution with the laser frequency. The color-coded traces in Fig. 3g plot the intensities at the marked positions in Fig. 3f as a function of the laser frequency change. All traces exhibit sinusoidal periodic evolutions with a common period of 173.4 GHz, which simply corresponds to the additional 804 ± 7 μm propagation length that the waveguide-coupled background experiences from the excitation site to GC1 (orange-dashed trace in Fig. 3a). These observations lead us to conclude that the background pattern variation is due to a simple two-part interference of waveguide-coupled and speckle-like scattered background fields. This finding and the measurements in Fig. 3g enable us to quantitatively determine the fraction of the waveguide-coupled background to be 6.5(3)% and thus the SBRs of 3320 ± 220 and 1660 ± 90 in the waveguide at the weak excitation limit and *S* = 1.0, respectively (Supplementary Note 6). Similar corresponding SBRs of 3610 ± 490 and 1810 ± 240 in the waveguide are obtained by studying the background properties from GC2 (Supplementary Note 6). We attribute such high SBRs to the following unique advantages of our platform. Both the AC nanosheet and nanofabricated Si_3_N_4_ waveguides have smooth surfaces with measured roughness of 0.2 nm in root-mean-square deviation (Supplementary Note 1). The naturally formed crystalline AC nanosheet possesses excellent mechanical properties and is free of cracks in the device even when the temperature is cooled to superfluid helium temperature. The controlled collective alignment of the molecular dipoles with the waveguide minimizes the excitation laser intensity. All these properties greatly suppress laser scattering into the waveguide mode.

### Ultrastable lifetime-limited molecular transition

**Fig. 4:**
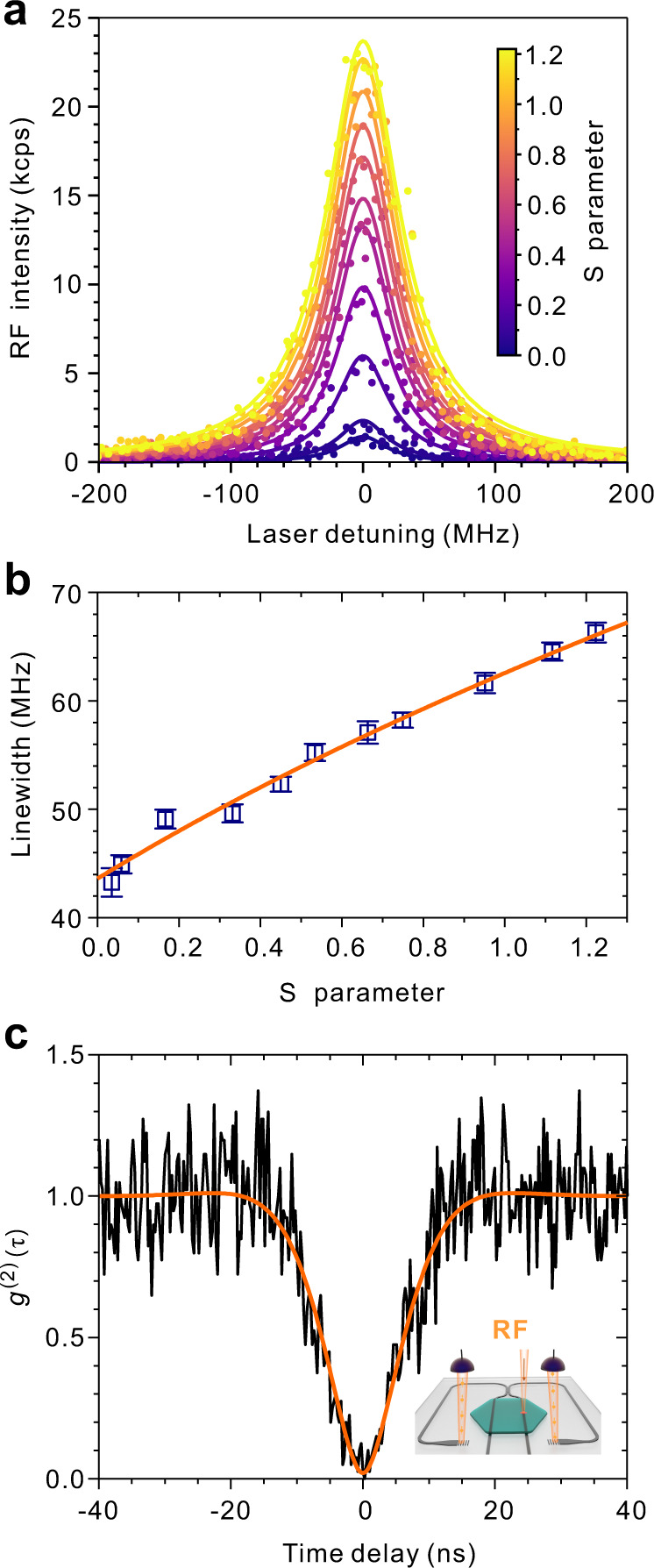
Lifetime-limited-linewidth transition and anti-bunching of RF photons that are beam split on the chip. **a** RF-excitation spectra at varied *S* (color-coded). The RF signals from GC1 and GC2 are combined and the background and dark count rates are subtracted. The laser frequency is scanned with a speed of 200 MHz/s. **b** Linewidth extracted from the measured RF-excitation spectra as a function of *S*. The solid orange line is the fit $\triangle \upsilon =\sqrt{1+S}/\left(\pi {\tau }_{2}\right)$. The error bars represent the fitting errors of the linewidths. **c** Second-order cross-correlation function (*g*^(2)^(*τ*)) of the RF photons from GC1 and GC2 when *S* = 0.64 (excitation power is 9.57 nW). The orange line is the theoretical curve for the RF (*g*^(2)^(0) = 0.020(1)) with the APD’s dark count considered. Inset: sketch of HBT experiment with on-chip beam splitting.

Next we examine if the linewidth of the molecular transition is lifetime-limited, i.e., $2{\tau }_{1}\cong {\tau }_{2}$, where *τ*_1_ and *τ*_2_ are the excited-state lifetime and decoherence time, respectively. Firstly we determine *τ*_2_ by studying the RF-excitation spectrum of a single molecule at varied laser excitation intensities as shown in Fig. 4a. According to the optical Bloch equation^43^, the linewidth ∆*υ* (in Hz) is related to the saturation parameter and decoherence time via $\triangle \upsilon =\sqrt{1+S}/\left(\pi {\tau }_{2}\right)$. Linewidths ∆*υ* of the RF-excitation spectra are extracted and plotted in Fig. 4b as a function of *S*. By fitting the ∆*υ* curve, we obtain the decoherence time of *τ*_2_ = 7.30 ± 0.09 ns and the linewidth at the weak excitation limit (i.e., *S*→0) of 43.61 ± 0.56 MHz. Excited-state lifetime *τ*_1_ can be extracted from the second-order photon correlation function *g*^(2)^(*τ*). Figure 4c presents *g*^(2)^(*τ*) of the RF photons split on the chip for the molecule under *S* = 0.64. A nearly perfect anti-bunching dip is again observed. By fitting *g*^(2)^(*τ*) with the consideration of APD dark count rate (Supplementary Note 7), we determine *τ*_1_ = 3.89 ± 0.38 ns. Therefore we achieve *τ*_2_/2*τ*_1_ = 0.94 ± 0.05, i.e., nearly lifetime-limited transition from a waveguide-coupled single molecule. This is a remarkable achievement for waveguide-coupled solid-state quantum emitters and is more stringent than the demonstration of subsequently emitted indistinguishable photons^21,22^.

**Fig. 5:**
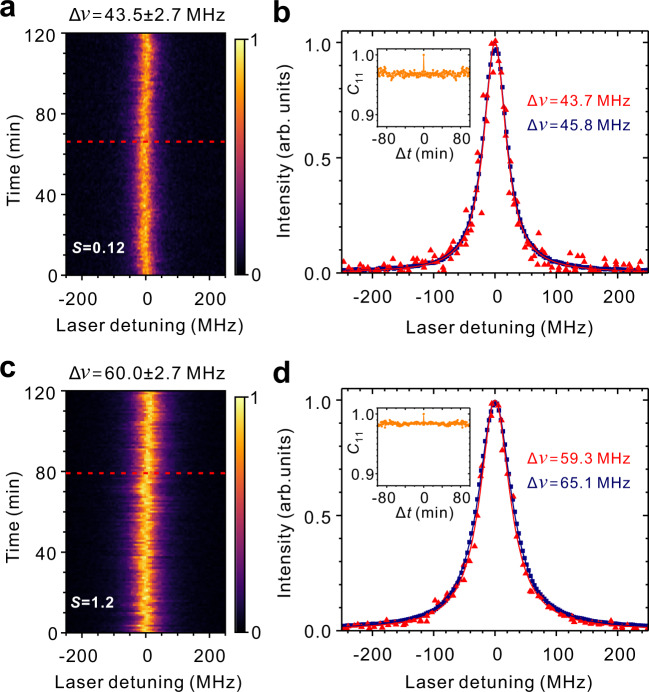
Spectral stability under resonant excitation. **a** Fluorescence-excitation spectra (*S* = 0.12) recorded for two hours with the excitation laser scanned across the 00ZPL at a speed of 200 MHz/s. **b** Superposition of all recorded fluorescence-excitation spectra (blue) and a typical single-scan fluorescence-excitation spectrum (red) recorded at the time marked by the red dashed line in **a**. Inset: normalized spectral autocorrelation as a function of time delay. **c**, **d** Same as **a**, **b**, respectively, but *S* = 1.2 (this value of *S* leads to a power broadening of the linewidth by 48%).

In the following, we demonstrate that the waveguide-coupled molecules can be almost free of spectral wandering for hours. Figure 5a presents the recorded fluorescence-excitation spectra under repeated laser scanning for two hours with a scan speed of 200 MHz/s at *S* = 0.12 for another molecule. These spectra possess a nearly lifetime-limited linewidth of 43.5 MHz with a standard deviation of 2.7 MHz. In Fig. 5b, the red symbols depict a typical single-scan spectrum while the blue symbols display the spectrum obtained from a direct superposition of all recorded excitation spectra. The latter represents an inhomogeneously broadened spectrum due to spectral diffusion over two hours and exhibits a linewidth of 45.8 MHz, which amounts to a broadening by only 2.3 MHz (*~*5%). The inset of Fig. 5b presents a spectral autocorrelation analysis, which indicates the correlation kept above 0.96 over the entire measurement time period. The spectral stability of the transition persists beyond the weak-excitation regime. As shown in Fig. 5c, d for the excitation intensity level of *S* = 1.2, the inhomogeneous broadening accumulated for two hours is as low as 8.5% and the spectral autocorrelation is kept above 0.98. Different molecules with similar spectral stability are shown in Supplementary Note 9. We remark that such level long-term spectral stability is not shared by any other waveguide-coupled solid-state quantum systems^16,18^. For quantum dots, the lifetime-limited linewidths are typically obtained from single-scan spectrums with a scanning frequency over 10 kHz (<0.1 ms per spectrum) to exclude effect of slow spectral diffusion^21^. Moreover, the spectral stability of our molecules is obtained without any feedback control for charge stabilization, which will become important when the system size scales up. The demonstration that the photonic-circuited molecules possess a lifetime-limited transition with such stability implies the generation of single-photon streams indistinguishable for hours and the prospect of overcoming the formidable challenge to have multiple emitters on one chip with a matched transition frequency.

We attribute the spectral stability to a series of advantages of our hybrid-integration platform. Spectral diffusion is often caused by nanoscopic charge fluctuations around the emitter from trapped or wandering charges around nanofabricated semiconductor surfaces^11,19,44^. The charge fluctuations could also be optically activated^33^ in particular for low bandgap materials. In our platform, the nanofabricated photonic circuits are based on wide-bandgap material Si_3_N_4_ and possess surfaces with a roughness of only 0.2 nm in root-mean-square deviation (Supplementary Note 1). The DBT molecules are embedded in the van der Waals bonded AC crystal^25,38^ which provides a stable host environment. Our crystalline AC nanosheets are free of nanofabrication and have smooth surfaces with relatively large areas (~10000 μm^2^) (Supplementary Note 1). A combination of these elements ensures a stable charge environment for the DBT molecules.

## Discussion

In summary, we have presented an organic-inorganic hybrid quantum photonic platform that enables on-chip generation, beam splitting and routing of background-free resonance fluorescence from single molecules with an ultrastable lifetime-limited transition. The organic part is nanofabrication-free and allows a collective alignment of the dipoles of the densely-doped molecules with the nanophotonic elements. The sample format also offers an almost independent design and fabrication of the inorganic part of the circuits. These advantages will readily allow the extension of the current photonic structures to include more complex architectures, for instance, in-plane microcavities to improve emitter-waveguide coupling efficiency from the current value of 8% (Supplementary Note 8) to near unity and to enhance the 00ZPL emission^14,15,27,45^, microelectrodes to electrically tune molecules’ transition via Stark effect for on-chip generation of indistinguishable single photons from independent molecules^46,47^, and superconducting nanowire single-photon detectors to benefit from the ultrahigh on-chip SBR^12^. The relatively large area (~10000 μm^2^) of the AC nanosheets in principle could simultaneously cover several tens of waveguides in parallel, which allow thousands of molecules simultaneously coupled to these waveguides in the circuit, promising a large scale integration. We remark that apart from the Si_3_N_4_ platform other platforms such as lithium niobate and aluminium nitride^7^, also could be employed to make properties of the nanophotonic elements electrically reconfigurable. Our molecular chip architecture together with the pick-and-place integration technique enables flexible interfacing with other on-chip systems such as color centers^10,16^, quantum dots^20^ and rare-earth ions^48^ to combine the merits of different systems. Thus we believe this work makes an important step to scalable molecular quantum photonics.

## Supplementary information

Supplementary Information

## Data Availability

The data that support the findings of this study are available from the corresponding authors upon reasonable request.

## Code availability

All codes used in this study are available from the corresponding authors upon reasonable request.
