# Supplementary material for: Photonic-circuited resonance fluorescence of single molecules with an ultrastable lifetime-limited transition
Source: Nat Commun. 2022 Jul 9;13:3982. doi: 10.1038/s41467-022-31603-x (PMC9271078; doi:10.1038/s41467-022-31603-x)
Supplement: Supplementary file 1 — Supplementary Information [file 41467_2022_31603_MOESM1_ESM.pdf]

Supplementary Information for

Photonic-circuited resonance fluorescence of single molecules with an ultrastable lifetime-limited transition

Penglong Ren[1,2†], Shangming Wei[1,2†], Weixi Liu[3†], Shupei Lin[1,2], Zhaohua Tian[1,2], Tailin Huang[1,2], Jianwei Tang[1,2*], Yaocheng Shi[3*] and Xue-Wen Chen[1,2*]

[1]School of Physics and Wuhan National Laboratory for Optoelectronics, Huazhong University of Science and Technology, Luoyu Road 1037, Wuhan, 430074, People's Republic of China

[2]Institute for Quantum Science and Engineering, Huazhong University of Science and Technology, Luoyu Road 1037, Wuhan 430074, People's Republic of China

[3]Centre for Optical and Electromagnetic Research, State Key Laboratory for Modern Optical Instrumentation, College of Optical Science and Engineering, Zhejiang University, Zijingang Campus, Hangzhou 310058, China

[†]These authors contributed equally to this work.

*To whom correspondence should be addressed: xuewen_chen@hust.edu.cn (X.-W.C.); jianwei_tang@hust.edu.cn (J.T.); yaocheng@zju.edu.cn (Y.S.)

Contents

Supplementary Note 1 – Fabrication and characterization of the hybrid quantum photonic circuits 4
a. Inorganic part: silicon-nitride based nanophotonic elements 4
i. Overview of the nanofabrication process 4
ii. Waveguides 4
iii. 2×2 MMI coupler as a beam splitter 5
iv. Grating couplers 6
b. Organic part: crystalline anthracene nanosheet with DBT molecules 6
i. Sample preparation: crystalline anthracene nanosheet 6
ii. Sample characterization: dimensions and surface morphology 7
c. Inorganic and organic hybrid integration with crystal orientation aligned 7
Supplementary Note 2 – Numerical simulations 10
a. Modal analysis of the hybrid waveguide structure 10
b. Coupling efficiency of a dipolar emitter to the waveguide modes 11
c. Emission patterns of the grating out-couplers in the Fourier plane 13
Supplementary Note 3 – Experimental setup 14
a. Module for controlling the power and polarization of the excitation laser 15
b. Excitation part 15
c. Detection part 16
Supplementary Note 4 – Fourier-plane imaging of the fluorescence signal and the laser background 17
Supplementary Note 5 – Laser background suppression in off-chip resonance fluorescence detection 18
a. Methods of laser-background suppression 18
b. Suppression factor of the laser background 18
c. Laser background and SBR measured at GC2 19
d. Laser background and SBR measured for single molecules at different locations 19
Supplementary Note 6 – Signal-to-background ratio of on-chip waveguided resonance fluorescence 21
a. Interference properties of laser background 21
b. Method to extract waveguide-coupled laser background from total background 21
c. Signal-to-background ratios of guided RF estimated from GC1 and GC2 22
Supplementary Note 7 – Determination of the excited-state lifetime 24
Supplementary Note 8 – Characterizations of RF saturation and coupling efficiency

into the waveguide **25**
a. RF saturation measurement 25
b. Grating coupling efficiency measurement 25
c. RF coupling efficiency into the waveguide mode 26
Supplementary Note 9 – Molecules with unprecedented spectral stability **27**
Supplementary References **28**

Supplementary Note 1 – Fabrication and characterization of the hybrid quantum photonic circuits

a. Inorganic part: silicon-nitride based nanophotonic elements

i. Overview of the nanofabrication process

Our planar photonic circuit is fabricated on the silicon nitride on insulator (SNOI) platform where the wafer consists of a 250 nm Si_3N_4 top layer, a 3 μm SiO_2 buffer layer and a silicon substrate. Firstly, the pattern of the photonic structures is defined on a negative resist (MaN-2403) using the electron beam lithography (EBL, Raith 150II). Then, inductively coupled plasma reactive ion etching (ICP-RIE) with mixed gases of CHF_3 and CF_4 is applied to transfer the resist pattern to the Si_3N_4 layer. In this work, the planar photonic circuit includes the following elements: Si_3N_4 waveguides, a 2×2 multi-mode interference (MMI) coupler and grating couplers. In the following, we provide more details about these photonic elements.

ii. Waveguides

Supplementary Fig. 1a and Fig. 1b display the scanning electron microscopy (SEM) images of a section of the fabricated bent Si_3N_4 waveguide in the oblique view and the cross section of the waveguide. Supplementary Fig. 1c shows an atomic force microscopy (AFM) topographic image for a part of the straight waveguide. A cross sectional plot along the blue dashed line in Supplementary Fig. 1c is depicted in Supplementary Fig. 1d, which indicates that the height of the waveguide is 270 nm due to a slight over etching of the SiO_2 buffer layer by 20 nm. Supplementary Fig. 1e presents a high-resolution AFM topographic image of the Si_3N_4 waveguide top surface within the region denoted by a dashed square box in Supplementary Fig. 1c. A typical line plot of the image is shown in Supplementary Fig. 1f. Based the high-resolution AFM measurement, the roughness of the waveguide top surface is estimated to be 0.2 nm in root-mean-square deviation, which is probably limited by the instrumental noise of our AFM.

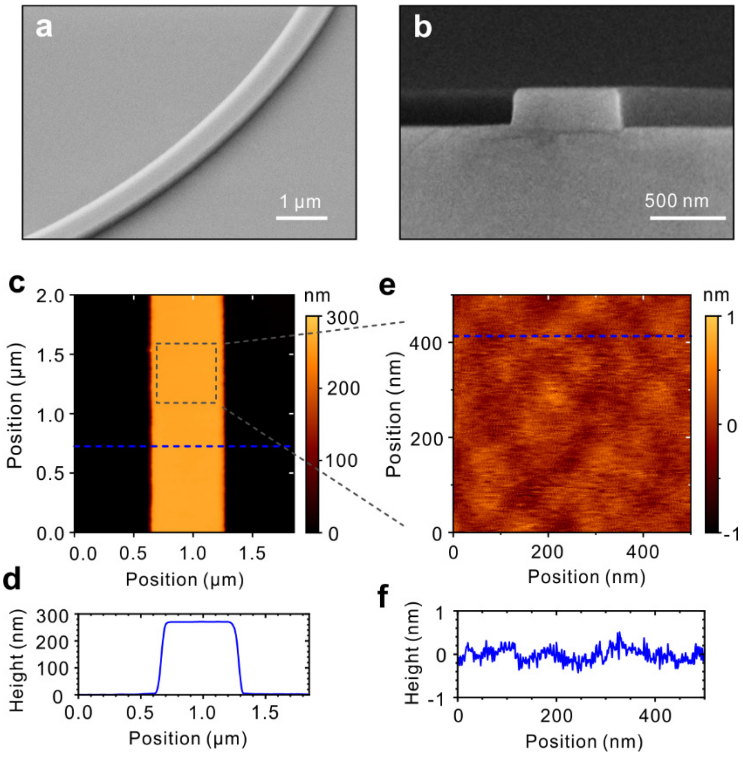

Supplementary Fig. 1 | SEM and AFM images of the fabricated Si_3N_4 waveguide. a, **b** SEM images of the fabricated Si_3N_4 waveguide in the oblique view (**a**) and in the cross sectional view (**b**). **c** AFM topographic image of the fabricated Si_3N_4 waveguide. **d** A cross sectional plot along the blue dashed line in **c**. **e** High-resolution AFM topographic image of the Si_3N_4 waveguide top surface for the region denoted in **c** by a dashed square box. **f** A cross sectional plot along the blue dashed line in **e**.

iii. 2×2 MMI coupler as a beam splitter

Supplementary Fig. 2a and 2b show the SEM images of the fabricated 2×2 MMI coupler in the vertical top view and oblique view, respectively. The length and width of the multimode waveguide are 14 μm and 1.8 μm, respectively. The gap between the two input/output waveguides is 140 nm.

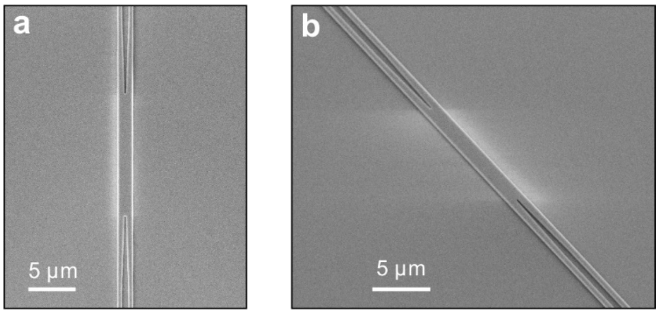

Supplementary Fig. 2 | SEM images of the fabricated 2×2 MMI coupler. a SEM image in the vertical top view. **b** SEM image in the oblique view.

iv. Grating couplers

Supplementary Fig. 3a and 3b show the SEM images of a fabricated grating coupler in the vertical top view and oblique view, respectively. The grating coupler has a width of 5 μm, a length of 15 μm, a period of 630 nm and a duty cycle of 0.72.

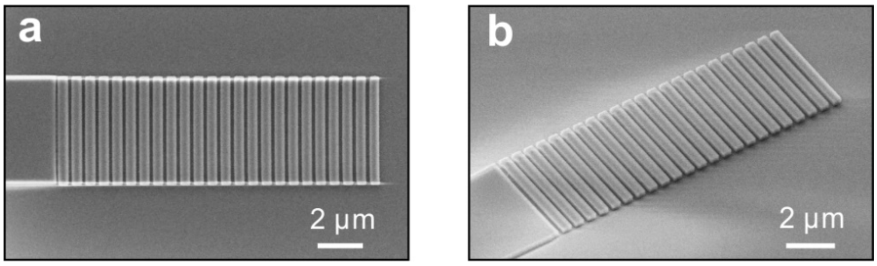

Supplementary Fig. 3 | SEM images of a fabricated grating coupler. a SEM image in the vertical top view. **b** SEM image in the oblique view.

b. Organic part: crystalline anthracene nanosheet with DBT molecules

i. Sample preparation: crystalline anthracene nanosheet

The crystalline anthracene (AC) nanosheets embedded with DBT molecules are obtained through a co-sublimation process[1]. Firstly, 7.8,15.16-dibenzoterrylene (DBT purity ~90%, from Chiron AS, Norway) and anthracene (purity ≥ 99%, from Sigma-Aldrich) are uniformly mixed with a mixing ratio of 1:500 by heating the mixture to 230°C (melted) in nitrogen atmosphere (30 kPa above standard atmospheric pressure) for one hour. Then, the mixture is cooled down to room temperature and 50 mg of the solid mixture is put into a glass tube and heated to 230°C in nitrogen atmosphere for 3 minutes with the tube closed. After that, the tube is unplugged to let the mixture vapor flow out of the tube and condense in the cool nitrogen atmosphere to form crystalline AC nanosheets with DBT molecules doped. A polyvinyl chloride (PVC) plastic substrate is used to collect the nanosheets floating in the nitrogen atmosphere. There are many DBT:AC nanosheets on the PVC substrate and one can freely select some of them for spectroscopic studies and applications. The nanosheets have excellent mechanical rigidity. We developed a transfer platform consisting of a tapered fiber tip mounted on 3D translation stage to pick up and transfer the nanosheets to other substrates.

ii. Sample characterization: dimensions and surface morphology

A typical optical micrograph of a DBT:AC nanosheet is shown in Fig. 1b. The nanosheet is in hexagonal shape and has an area of 7200 μm^2. Supplementary Fig. 4a shows an AFM topographic image for a part of the nanosheet. Supplementary Fig. 4b depicts a cross sectional plot along the dashed line in Supplementary Fig. 4a and indicates that the nanosheet has a thickness of 150 nm. About 5 µm away from the edges, the surface of the crystalline nanosheet is already very flat. Supplementary Fig. 4c shows a high-resolution AFM topography image within the region denoted by a dashed square box in Supplementary Fig. 4a. From the high-resolution image, the roughness is estimated to be 0.2 nm in root-mean-square deviation, which is probably limited by the instrumental noise of our AFM.

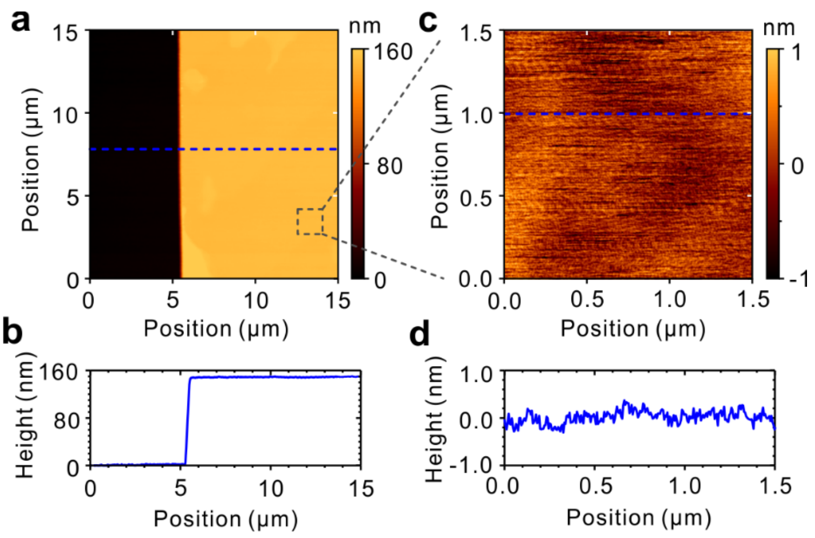

Supplementary Fig. 4 | AFM images of the crystalline AC nanosheet. a AFM topographic image for a part of the crystalline nanosheet. **b** A cross sectional plot of along blue dashed line in **a**. **c** High-resolution AFM topographic image of the surface of the nanosheet for the region denoted in **a** by a dashed square box. **d** A cross sectional plot of along blue dashed line in **c**.

c. Inorganic and organic hybrid integration with crystal orientation aligned

The large lateral size and the mechanical rigidity of the nanosheet enables us to hybrid integrate with the inorganic SNOI photonic circuit through a pick-and-place procedure. Moreover, the hexagonal shape of the nanosheet allows us to identify the *b*-axis of the AC crystal and to align it with the electric field of the fundamental TE mode of the waveguide. Since emission dipole moments of the embedded DBT molecules collectively orient along the

b-axis of the AC crystal, the platform allows an optimal alignment between the photonic waveguide and the molecules in a collective manner.

The hybrid integration procedure is sketched in Supplementary Fig. 5 and described as follows:

a) Under an optical microscope we select a suitable nanosheet from the nanosheet reservoir collected on a PVC substrate and a tapered fiber tip mounted on a 3-dimensional translation stage is gently pressed onto a corner of the nanosheet and then lifted to pick up the nanosheet (Supplementary Fig. 5a).

b) The chip with the photonic circuit is placed on the microscope stage and the picked-up nanosheet is brought closer to the chip surface so that both the nanosheet and the waveguides are within the depth of focus of the microscope (Supplementary Fig. 5b).

c) The chip is rotated and translated so that the target region of waveguide is right under the nanosheet and orthogonal to the "*b*" axis of the crystalline nanosheet (Supplementary Fig. 5c).

d) The nanosheet is brought even closer to the chip surface until it contacts the waveguide. Once the nanosheet contacts the chip surface, it immediately attaches tightly to the surface via van der Waals forces and detaches from the fiber tip (Supplementary Fig. 5d).

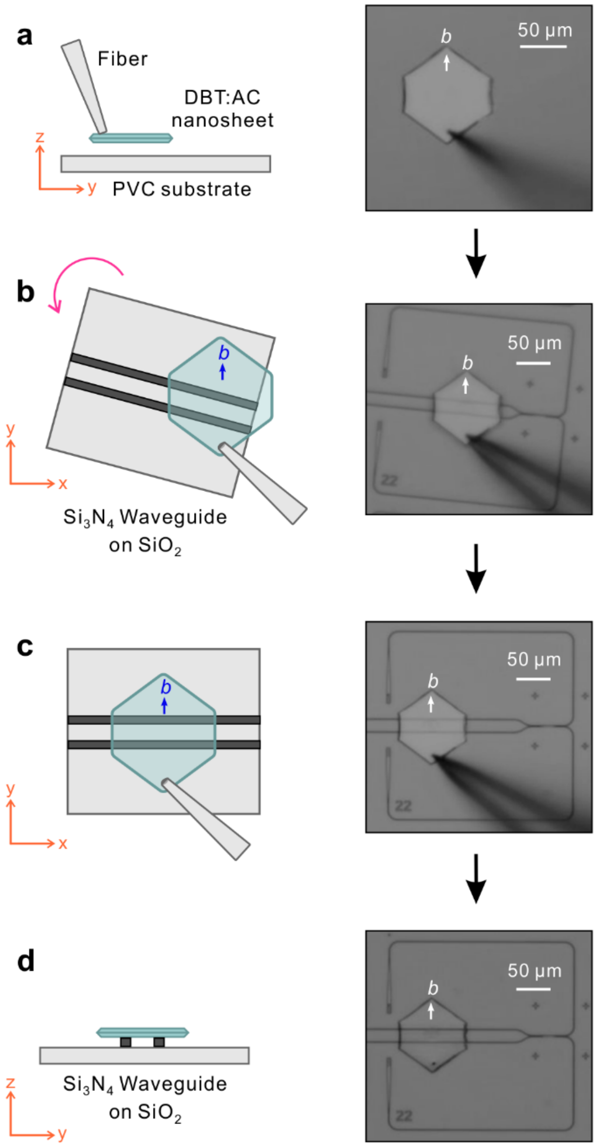

Supplementary Fig. 5 | Pick-and-place process for hybrid integration of a DBT-doped crystalline AC nanosheet with the photonic circuit. a A suitable nanosheet is selected from the nanosheets collected on a PVC substrate and a fiber tip is pressed onto a corner of the nanosheet and then lifted to pick up the nanosheet. Right panel: Optical micrograph of a nanosheet picked up by the fiber tip. **b** The picked-up nanosheet is brought close to the chip surface. Right panel: Optical micrograph of a nanosheet close to the chip surface. **c** The chip is rotated and translated to align the waveguide with the nanosheet. Right panel: Optical micrograph of a nanosheet aligned with the waveguide. **d** The nanosheet attaches to the surface via van der Waals forces and detaches from the fiber tip. Right panel: Optical micrograph of the final hybrid integrated quantum photonic circuit. There is a small defect left at the contact position with the tapered fiber tip.

Supplementary Note 2 – Numerical simulations

Lumerical FDTD Solutions are used for full-wave electromagnetic simulations and Lumerical Mode Solutions are used for the waveguide modal analysis. In the numerical simulations, the refractive indices of Si_3N_4 and SiO_2 buffer layer are set to be 2.02 and 1.46, respectively. Although the crystalline anthracene is a biaxial anisotropic material[2-5], here considering that the "*b*" axis of crystalline anthracene is aligned with the electric field of TE mode of the waveguide, anthracene is simply approximately modelled as having an isotropic refractive index of 1.8. This is the refractive index along the "*b*" axis of crystalline anthracene[2-5]. One may note that although the literature only provided the refractive index at wavelengths below 600 nm, we take it for our calculation around 780 nm because according to the literature[3] single-crystalline anthracene has no absorption peaks for light with wavelength longer than 420 nm and the dispersion curves are almost flat at wavelength longer than 500 nm.

a. Modal analysis of the hybrid waveguide structure

Supplementary Fig. 6a (6g) shows the cross-sectional structure of the waveguide without (with) the AC nanosheet on top. The Si_3N_4 waveguide height is 250 nm. The thickness of the AC nanosheet is 150 nm. The 20 nm over-etching of the SiO_2 buffer layer is also considered. Supplementary Fig. 6b (6h) plots the effective index n_{eff} of the guided modes of the lowest orders as a function of the waveguide width without (with) the AC nanosheet on top. We find that for a waveguide width below 600 nm (which is the width for our fabricated sample), only the fundamental quasi-TE mode (denoted as TE_0) and the fundamental quasi-TM mode (denoted as TM_0) are supported, whether the waveguide is covered by the AC nanosheet or not. Supplementary Fig. 6c,d (i,j) show the electric field distribution of the TE_0 mode for the waveguide without (with) the AC nanosheet on top when the waveguide width is set to be 600 nm. We observe that with the AC nanosheet on top, the mode profile is slightly pulled towards the AC nanosheet, which is favorable for the DBT molecules in the AC nanosheet to couple to the guided mode. Supplementary Fig. 6e,f (k,l) show the electric field distribution of the TM_0 mode for the waveguide without (with) the AC nanosheet on top.

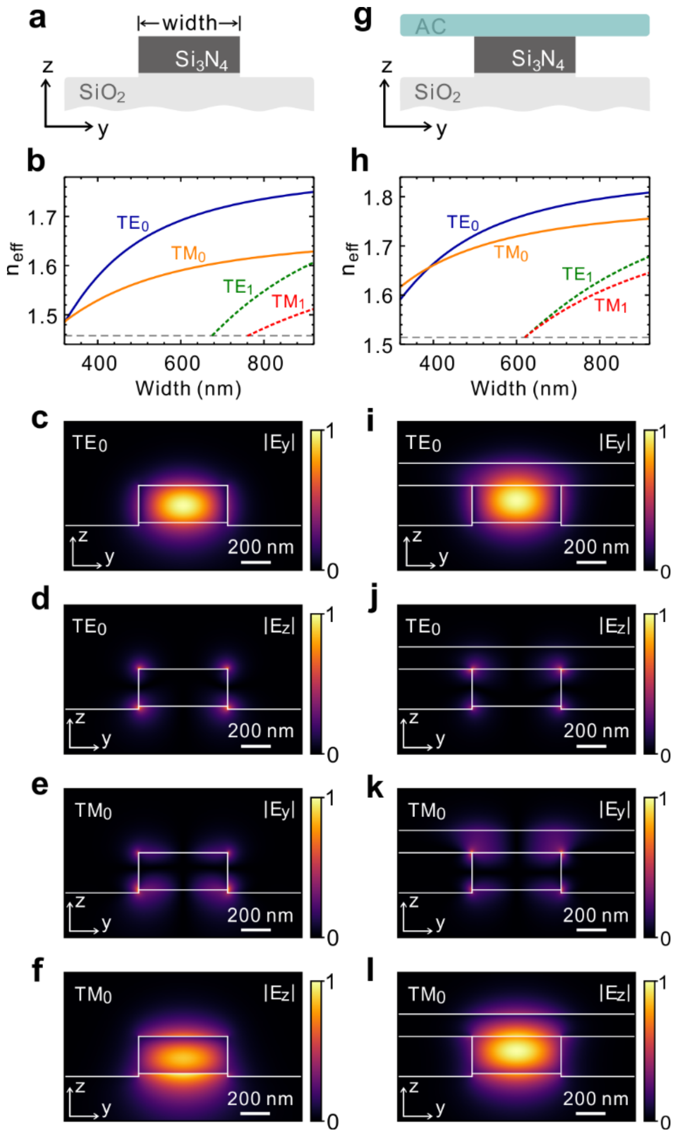

Supplementary Fig. 6 | Modal analysis of the waveguides. a Cross-sectional structure of the waveguide without an AC nanosheet on the top. The 20 nm over etching of the SiO_2 buffer layer is considered. **b** Effective index n_{eff} of the guided modes of the lowest orders as a function of the waveguide width without the AC nanosheet on top. **c-f** Distribution of $|E_y|$ (**c**,**e**) and $|E_z|$ (**d**,**f**) of the TE_0 (**c**,**d**) and TM_0 (**e**,**f**) mode for the waveguide without the AC nanosheet on top when the waveguide width is 600 nm. **g-l** Same as **a-f**, but the waveguide is covered by an AC nanosheet of 150 nm in thickness.

b. Coupling efficiency of a dipolar emitter to the waveguide modes

The coupling efficiency $\beta_m^{\pm}$ of a molecule to a target waveguide mode m propagating in the $\pm x$ direction is the ratio between the emission rate into the target waveguide mode $\Gamma_m^{\pm}$ and the total emission rate Γ_{tot}, i.e.,

$$\beta_m^{\pm} = \frac{\Gamma_m^{\pm}}{\Gamma_{\text{tot}}} \, . \tag{1}$$

The vacuum-normalized emission rate $\Gamma_m^{\pm}/\Gamma_0$ is calculated as[6,7]

$$\frac{\Gamma_m^{\pm}}{\Gamma_0} = \frac{1}{2} \cdot \frac{3\pi c \varepsilon_0}{k_0^2} \frac{[\mathbf{E}_m^{\pm}(\mathbf{r}_0) \cdot \mathbf{n}_\mathrm{d}][\mathbf{E}_m^{\pm *}(\mathbf{r}_0) \cdot \mathbf{n}_\mathrm{d}]}{\iint (\mathbf{E}_m^{\pm} \times \mathbf{H}_m^{\pm *}) \cdot (\pm \mathbf{n}_x)\, \mathrm{d}y\mathrm{d}z}, \quad (2)$$

where Γ_0 is the emission rate in vacuum, $\mathbf{n}_\mathrm{d}$ is a unit vector describing the dipole orientation (here it is in the *y* direction), $\mathbf{r}_0$ is the position of the dipole, $\mathbf{n}_x$ is a unit vector in the *x* direction, $\mathbf{E}_m^{\pm}$ and $\mathbf{H}_m^{\pm}$ are the electric- and magnetic-field vectors of guided mode *m* propagating in the $\pm x$ direction. $\mathbf{E}_m^{\pm}$ and $\mathbf{H}_m^{\pm}$ are obtained by performing a 2D mode analysis based on Lumerical Mode Solutions.

The vacuum-normalized total emission rate $\Gamma_{\mathrm{tot}}/\Gamma_0$ is obtained by performing a 3D electromagnetic simulation and calculating the ratio between the radiation power of the dipole coupled with the waveguide and the radiation power of the same dipole in vacuum. The radiation power is calculated by integrating the Poynting vector over a closed surface that encloses the dipole. With $\Gamma_m^{\pm}/\Gamma_0$ and $\Gamma_{\mathrm{tot}}/\Gamma_0$ calculated, $\beta_m^{\pm}$ is then obtained via Supplementary Equation (1). In our study the dipole couples equally to the forward and backward directions and therefore the coupling efficiency to guided mode *m* is $\beta_m = \beta_m^+ + \beta_m^-$. The coupling efficiency is a function of the dipole position. Supplementary Fig. 7a shows the coupling efficiency to TE_0 mode for dipole positions within the region above the waveguide. The coupling efficiency β_m for a molecule at the center of the AC nanosheet is 12.4% and the maximum value could reach 27.5%. Supplementary Fig. 7b shows the coupling efficiency to the TM_0 mode. One sees that the coupling to TM_0 mode is negligible as compared with TE_0 mode.

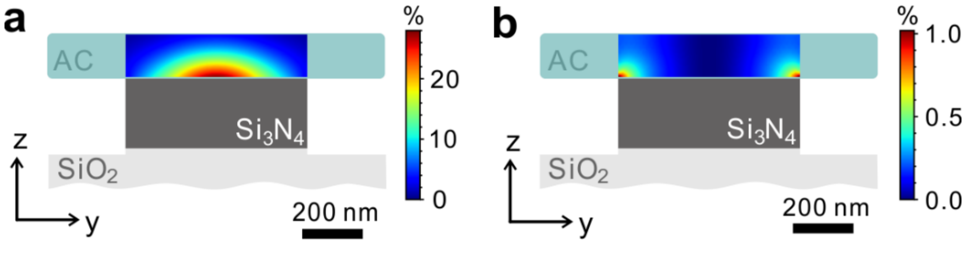

Supplementary Fig. 7 | Coupling efficiency of dipole emission into the waveguide mode. a, **b** Coupling efficiency of dipole emission to the TE_0 (**a**) and TM_0 (**b**) mode for a *y*-oriented (i.e., oriented along the "*b*" axis of the AC crystal) linear electric dipole as a function of dipole position.

c. Emission patterns of the grating out-couplers in the Fourier plane

The grating coupler has a width of 5 µm, a length of 15 µm, a period of 630 nm and a duty cycle of 0.72. To simulate the far-field pattern of the output from the grating coupler, we perform 3D FDTD simulations. An excitation source with the TE_0 waveguide mode profile is placed at the cross section of the waveguide which connects the grating coupler. The near-field distribution over a plane slightly above the grating coupler is recorded. Then a near-to-far field transformation is performed to obtain the far field distribution[8], which is then converted to the Fourier plane image of the microscopy system[9]. The simulated Fourier-plane pattern for GC1 (GC2) is shown in Supplementary Fig. 8a (8b), which agrees with the measured Fourier plane image shown in Supplementary Fig. 8b (8d). Both the measured pattern and simulated pattern show that the output angle of the grating coupler is ~16.5°.

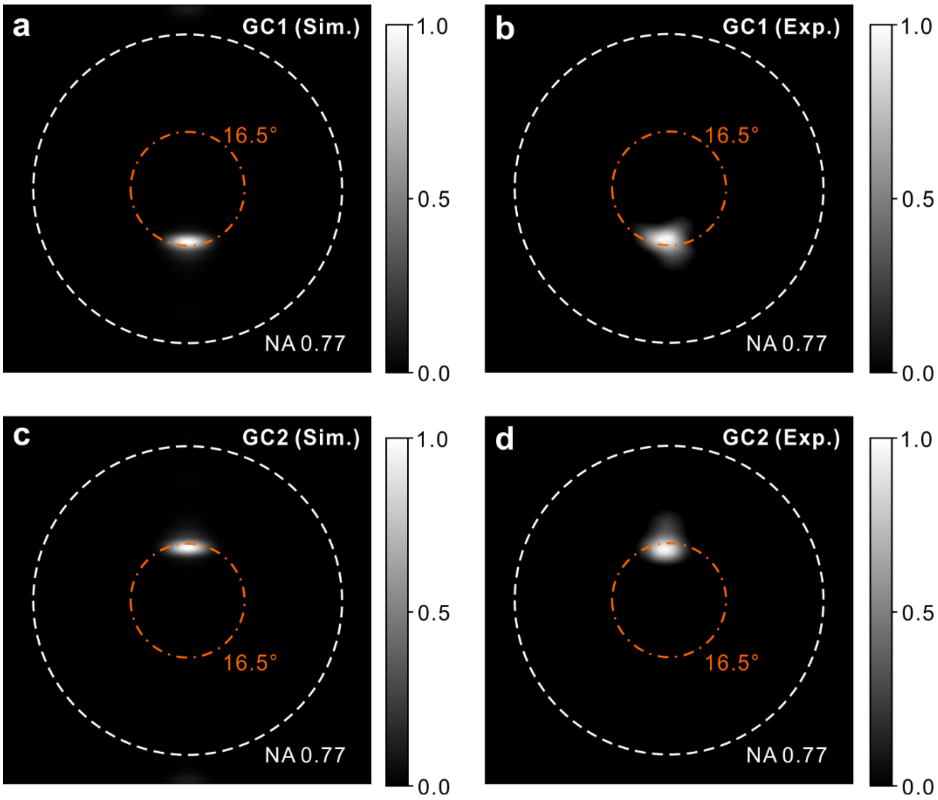

Supplementary Fig. 8 | Fourier-plane pattern of the radiation from grating couplers. a, **b** Simulated (**a**) and measured (**b**) Fourier-plane patterns of the output from GC1. **c**, **d** Simulated (**c**) and measured (**d**) Fourier-plane patterns of the output from GC2. The white dashed circle denotes the value of the numerical aperture (NA) of our aspherical lens (0.77). The orange dash-dotted circle denotes an output angle of 16.5°.

Supplementary Note 3 – Experimental setup

The full experimental setup for optical measurements is shown in Supplementary Fig. 9. The setup consists of three major parts as indicated by the color-coded shadings. The part in blue-shaded region is an optics module to stabilize and control the intensity and polarization state of the excitation source. The green-shaded and red-shaded regions are the excitation and detection parts of the optical setup, respectively. The three parts are described as follows.

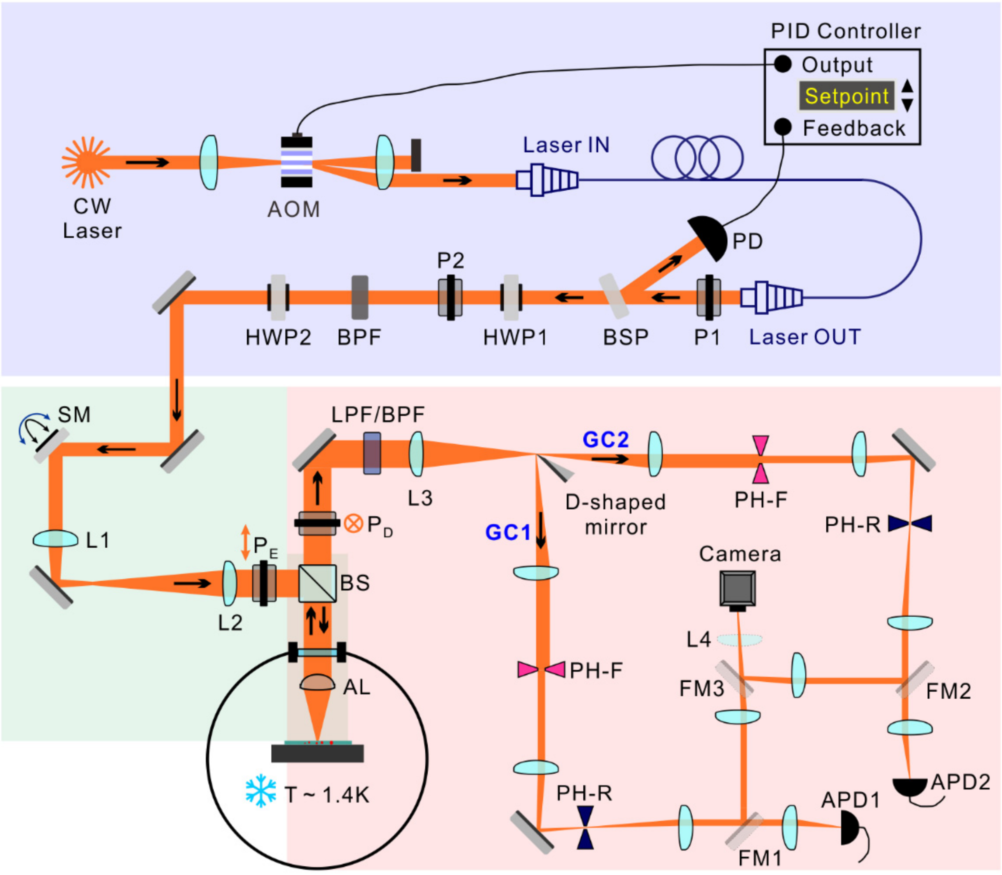

Supplementary Fig. 9 | Full experimental setup for optical measurement. The part in the blue-shaded region is a module of optics to stabilize and control the intensity and polarization state of the excitation source. The green-shaded and red-shaded regions are the excitation and detection parts of the optical setup, respectively. The sample is placed in a Helium Cryostat (T ~ 1.4 K) indicated by a circle. SM: steering mirror; BS: beamsplitter; BSP: beam sampling plate; AL: aspheric lens; $P_{E/D}$: polarizer for excitation/detection; PH-F: Fourier-plane pinhole; PH-R: real-image-plane pinhole; LPF: long-pass filter; BPF: band-pass filter; APD: avalanche photodiode single photon detector; AOM: acousto-optic modulator; PD: photodiode; P1, P2: polarizers; HWP: halfwave plate; FM: flip mirror.

a. Module for controlling the power and polarization of the excitation laser

The excitation laser source is a tunable CW single-mode laser system (Matisse 2TS, Spectra-Physics) that is actively stabilized with an external reference cavity to make the laser linewidth below 50 kHz. The output light from the laser system is coupled into a polarization maintaining single-mode fiber and power-stabilized via a closed-loop feedback system. The closed-loop feedback system is as follows: Firstly, the output light from the laser system is focused by a lens to an acoustic optical modulator (AOM) and the diffracted beam is re-collimated by a lens and coupled into a polarization maintaining single-mode fiber; then the light output from the other end of the polarization maintaining single-mode fiber is collimated; a linear polarizer (P1) is used to improve the degree of linear polarization; then a small part of the light is reflected by a beam sampling plate (BSP) and detected by a photodiode (PD); the electronic signal of the photodiode is sent as a feedback signal to a PID controller; the output of the PID controller is sent to the AOM to control the diffraction efficiency of the beam so that the power detected at the photodiode is stabilized and consequently the power of the light beam after the linear polarizer P1 is stabilized. After the power stabilization, the power fluctuation is suppressed to below 0.5%. The power-stabilized light beam then successively passes through a half-wave plate (HWP1) and a linear polarizer (P2) so that the light power is continuously tunable. A half-wave plate (HWP2) is then used to control the polarization angle.

b. Excitation part

After the above module, the excitation laser light beam is reflected by a pair of steering mirrors (SM) for realizing beam scanning over the sample. The beam passes through a pair of lenses L1 and L2, which form a 4f optical system to conjugate the steering mirrors to the back focal plane of the aspheric lens (AL; NA=0.77). After passing through the lenses L1 and L2, the beam is reflected by a non-polarizing cube beamsplitter (BS) with a splitting ratio of 10:90 (R:T) and subsequently focused onto the sample by the aspheric lens (AL). By controlling the steering mirrors (SM), the position of the focal spot at the sample can be controlled. The aspheric lens (AL) is mounted on a piezoelectric nanopositioner (ANPx51, Attocube) for

flexible adjustment of the focus in the axial direction. The sample is placed on a 2D piezoelectric nanopositioner (ANPx51 and ANPz51, Attocube) to realize two-dimensional adjustment of the sample position with respect to the laser beam.

c. Detection part

The optical signals from the sample are first collected by the aspheric lens (AL) and then passes through the beam splitter (BS). Then after lens L3 the first real-plane image is formed. At this real image plane, a D-shaped mirror is used to split the field of view, with the grating coupler GC1 in one half and GC2 in the other half, each into a separate light path (denoted as GC1 and GC2 light paths). In each light path, GC1 or GC2 is further conjugated to an avalanche photodiode single photon detectors (APD) via cascaded 4f systems. A pinhole at the Fourier plane (PH-F) and a pinhole at the real plane (PH-R) are used for Fourier-plane spatial filtering and real-plane spatial filtering, respectively. The pinholes are rectangular and their length, width and position are tunable. By flipping the mirror FM1 into the light path, the GC1 light can be directed to the scientific CMOS camera (orca-flash4.0, Hamamatsu) for imaging. By flipping the mirrors FM2 and FM3 into the light path, the GC2 light can be directed to the scientific CMOS camera for imaging. The images could be used to analyze the properties of the laser background and resonant fluorescence signal.

Supplementary Note 4 – Fourier-plane imaging of the fluorescence signal and the laser background

For Fourier-plane imaging, the Fourier planes of the light output from GCs are conjugated to the camera plane by flipping lens L4 into the light path at an appropriate position (see the red-shaped part in Supplementary Fig. 9). To obtain the Fourier-plane image of the waveguided signal output from the grating coupler, *i.e.*, Fig. 3b of the main text, the molecule is excited according to $|g,0\rangle \rightarrow |e,1\rangle$ transition (*i.e.*, 0-1 excitation) and the laser background is spectrally removed with a band-pass filter. To obtain the Fourier-plane image of the laser background, *i.e.*, Fig. 3c of the main text, the excitation laser beam is placed at the same position with its frequency detuned from the molecule's 00ZPL by 200 GHz. Note that for both Fig. 3b and 3c, the real-plane spatial filtering and cross-polarization filtering are applied, *i.e.*, the pinhole in the real plane (PH-R) is in position and the polarizer in the detection path (P_D) (see methods of laser-background suppression in Supplementary Section 5a) is set to be orthogonal to the excitation polarization. The camera works in sequence acquisition mode and the exposure time for every frame is set to be 10 seconds. Ten consecutive frames are superimposed to obtain the images shown in Fig. 3b and 3c of the main text.

Supplementary Note 5 – Laser background suppression in off-chip resonance fluorescence detection

a. Methods of laser-background suppression

To suppress the laser background, three methods are combined. Firstly, we bend the waveguide in such a way that the polarization of the output signal is orthogonal to the excitation laser. Since the laser polarization is aligned with the electric field of the fundamental TE mode (TE_0) of the waveguide, we can apply a cross-polarization detection technique by placing a polarizer orthogonal to the excitation polarization in the detection path (P_D), which suppresses the excitation laser without blocking any RF signal. Secondly, since the RF signal output from the grating has a narrow distribution in the Fourier plane (Fig. 3b of the main text) while the laser background shows a speckle-like and broad distribution in the Fourier plane (Fig. 3c of the main text), we place a rectangular pinhole in the Fourier plane (PH-F) to block a large portion of the laser background and allow the RF signal to pass. Besides applying the two above measures, we realize that the excitation and collection spots are spatially separated by 50~80 μm (depending on the exact position of the molecule) and thus apply a spatial filtering to further suppress the laser background. Specifically, a rectangular pinhole in real image plane (PH-R) is used to allow only the region of the grating coupler to be imaged to the detector.

b. Suppression factor of the laser background

The combination of the above three methods finally allow us to suppress the laser background by more than 7 orders of magnitude without significantly reducing the RF signal. The suppression factor of the laser background f is obtained by comparing the directly detected background intensity (without applying the above three methods) and the background intensity with the above three methods applied. In experimental measurements, the laser frequency is detuned from the molecule transition 00ZPL by 200 GHz for the generation of laser background without producing any fluorescence signal. For the measurement of the laser background without applying the above methods (*i.e.*, the polarizer P_D, PH-F and PH-R are all flipped out of the detection light path), the light is attenuated by a neutral-density filter with known transmittance to avoid saturating the detectors. The measured background suppression factor is 3.0×10^{-8} (5.6×10^{-8}) when the detection port is GC1 (GC2).

c. Laser background and SBR measured at GC2

The power-dependent laser background and SBRs from GC1 are shown in Fig. 3e of the main text. Here we present the above quantities of the output light from GC2 for the same molecule in Supplementary Fig. 10. By comparing with the results from GC1, we observe that the background from GC2 is a bit higher and thus the SBR is lower. This is understandable because the distance between GC2 and the excitation spot is shorter than the distance between GC1 and the excitation point. As indicated in Fig. 1a of the main text, the studied molecule locates at WG_{i2}, which is closer to GC2 than to GC1 by about 15 μm.

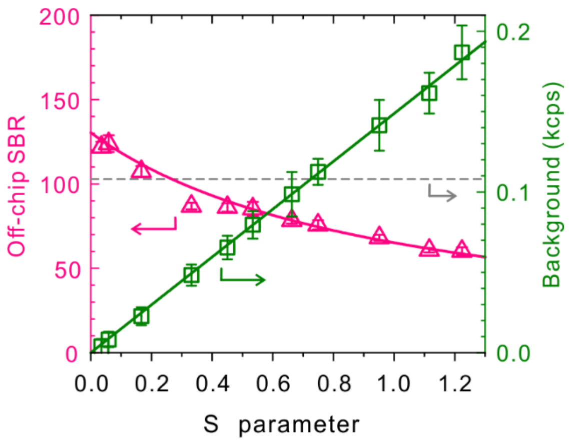

Supplementary Fig. 10 | Background and SBR measured at GC2. Pure background count rates (the dark count excluded) (green, right y axis) and SBR (pink, left y axis) as functions of the saturation parameter *S*. Green squares represent the measured pure background count rates (error bars represent the standard deviations). Green solid line is a linear fit. Pink triangles represent the measured SBRs (error bars stem from the fitting errors in the measured RF count rates). Pink solid line displays the calculated SBR from the RF saturation curve (Supplementary Note 8). The gray dashed line denotes the measured dark count rate of APD 2. Note that the measured dark count rates of APD1 (Fig. 3e of the main text) and APD2 have the same mean value of 108 counts per second.

d. Laser background and SBR measured for single molecules at different locations

Supplementary Fig. 11 displays the RF excitation spectra of a series of single molecules at different locations of the waveguide. The measurements are performed for emission from GC1. Since the molecules are addressed by steering the direction of the laser beam, the background is slightly different. Nevertheless, here an average of SBR of ~90 is routinely obtained.

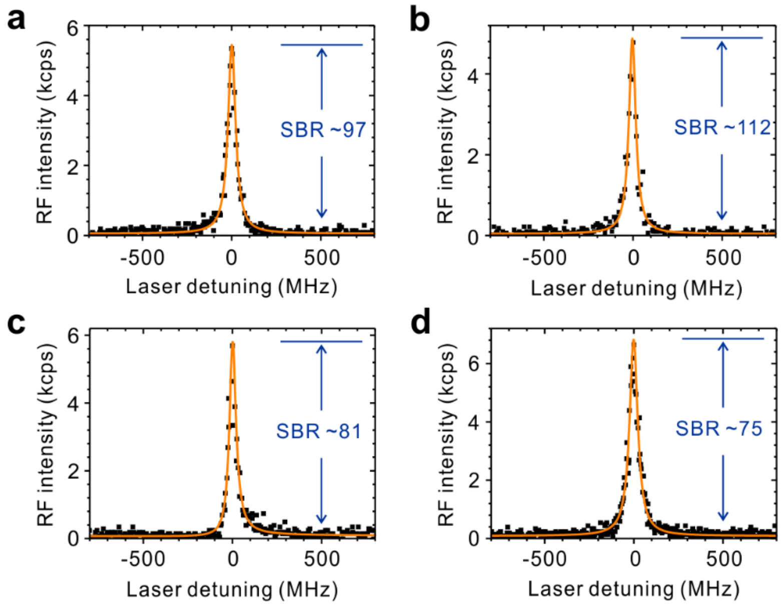

Supplementary Fig. 11 | RF excitation spectra of a series of single molecules at different locations. a RF excitation spectrum of a single molecule detected from GC1. For calculating the SBRs, dark count rates are subtracted from the background count rates. **b**, **c**, **d** Same as **a**, but the single molecules are at different locations.

Supplementary Note 6 – Signal-to-background ratio of on-chip waveguided resonance fluorescence

a. Interference properties of laser background

In this subsection, on top of the discussion associated with Fig. 3f and 3g, we provide some additional quantitative support to the fact that the total laser background pattern $\left|\mathbf{E}_{\mathrm{b,tot}}\right|^2$ is an interference between the waveguide-coupled background field $\mathbf{E}_{\mathrm{b,wg}}$ and the directly scattered speckle-like background field $\mathbf{E}_{\mathrm{b,sp}}$. All the traces in Fig. 3g of the main text exhibit sinusoidal periodic evolutions with a common period of Δf =173.4 GHz, which indicates that the two fields $\mathbf{E}_{\mathrm{b,wg}}$ and $\mathbf{E}_{\mathrm{b,sp}}$ should have an optical group delay of $\tau_{\mathrm{g}} =$ 5.8 ps. This is just expected, since, as compared with $\mathbf{E}_{\mathrm{b,sp}}$, $\mathbf{E}_{\mathrm{b,wg}}$ experiences an additional propagation path of L_{wg} = 804±7 μm through the Si_3N_4 waveguide from the excitation point to the grating out-coupler (see orange arrows in Fig. 3a of the main text), which amounts to a group delay of $\tau_{\mathrm{g,wg}}$ = 5.69±0.05 ps with the consideration of a group index of 2.12. The two numbers (τ_{g}, $\tau_{\mathrm{g,wg}}$) agree well and confirm the physical picture of two-field interference.

b. Method to extract waveguide-coupled laser background from total background

$\mathbf{E}_{\mathrm{b,wg}}$ is linearly polarized and aligned with polarizer P_D in the light path. $\mathbf{E}_{\mathrm{b,sp}}$ passes the same polarizer. Thus the total background field is a result of interference of two scalar fields. Consequently, from the color-coded interference trace for each sampling position in Fig. 3g of the main text, we have

$$I_{\max}(x_i, y_i) = \left(\left|\mathbf{E}_{\mathrm{b,sp}}(x_i, y_i)\right| + \left|\mathbf{E}_{\mathrm{b,wg}}(x_i, y_i)\right|\right)^2, \quad (3)$$

$$I_{\min}(x_i, y_i) = \left(\left|\mathbf{E}_{\mathrm{b,sp}}(x_i, y_i)\right| - \left|\mathbf{E}_{\mathrm{b,wg}}(x_i, y_i)\right|\right)^2, \quad (4)$$

where (x_i, y_i) is the corresponding marked position, $I_{\max}(x_i, y_i)$ and $I_{\min}(x_i, y_i)$ are the maximum and minimum values of the (fitted) sinusoidal curve, respectively. The above equations enable us to obtain a solution of $\left|\mathbf{E}_{\mathrm{b,wg}}(x_i, y_i)\right| = \frac{1}{2}\left(\sqrt{I_{\max}(x_i, y_i)} - \sqrt{I_{\min}(x_i, y_i)}\right)$ or $\frac{1}{2}\left(\sqrt{I_{\max}(x_i, y_i)} + \sqrt{I_{\min}(x_i, y_i)}\right)$ for point (x_i, y_i). The crucial condition is the fact that $\left|\mathbf{E}_{\mathrm{b,wg}}(x_i, y_i)\right|^2$ at all the sampling positions are related through one fixed radiation pattern of the grating coupler $|\mathbf{E}_{\mathrm{GC}}|^2$, which is experimentally measured and shown in Supplementary Fig. 12. By considering solutions of Supplementary Equations (3) and (4)

for all the sampling positions for satisfying this condition, we are able to remove the ambiguity for each position.

Knowing $|\mathbf{E}_{\mathrm{b,wg}}(x_i, y_i)|$ at all the sampling positions and the profile $|\mathbf{E}_{\mathrm{GC}}|^2$, we then calculate the fraction of the waveguide-coupled background in the total background as

$$\eta_{\mathrm{b,wg}} = \frac{|\mathbf{E}_{\mathrm{b,wg}}(x_i, y_i)|^2}{|\mathbf{E}_{\mathrm{GC}}(x_i, y_i)|^2} \frac{\iint |\mathbf{E}_{\mathrm{GC}}|^2 \mathrm{d}x\mathrm{d}y}{\iint |\mathbf{E}_{\mathrm{b,tot}}|^2 \mathrm{d}x\mathrm{d}y}, \quad (5)$$

for all the sampling positions.

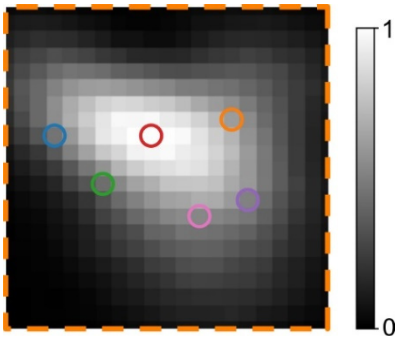

Supplementary Fig. 12 | Radiation pattern of GC1. This pattern is the Fourier-plane image of the part passing through the pinhole (PH-F), i.e., the region enclosed by the dashed rectangle in Fig. 3b of the main text. The colored circles are the sampling positions.

c. Signal-to-background ratios of guided RF estimated from GC1 and GC2

For each sampling position (x_i, y_i), the obtained $\eta_{\mathrm{b,wg}}$ is displayed along with the corresponding color-coded trace in Fig. 3g. The values of $\eta_{\mathrm{b,wg}}$ consistently turn out to be 6.5(3)%, *i.e,* the SBR in the waveguide is estimated to be more than an order higher than the SBR measured via off-chip detection. Considering that off-chip SBRs of 216 and 108 are obtained at the weak excitation limit and S = 1.0, respectively, SBRs of 3320$\pm$220 and 1660$\pm$90 are anticipated in the waveguide at the weak excitation limit and S = 1.0, respectively. The contribution of the waveguide-coupled laser background is also estimated from GC2 using the same procedure. The relevant data is shown in Supplementary Fig. 13. The value of $\eta_{\mathrm{b,wg}}$ estimated from GC2 turns out to be 3.6(4)%. Considering that for GC2, off-chip SBRs of 130$\pm$6 and 65$\pm$2 are obtained at the weak excitation limit and S = 1.0, respectively, SBRs in the waveguide are estimated to be 3610$\pm$490 and 1810$\pm$240 at the weak excitation limit and S = 1.0, respectively. As expected, the SBRs in the waveguide estimated from GC1 and GC2 are similar, which also serves as a cross check.

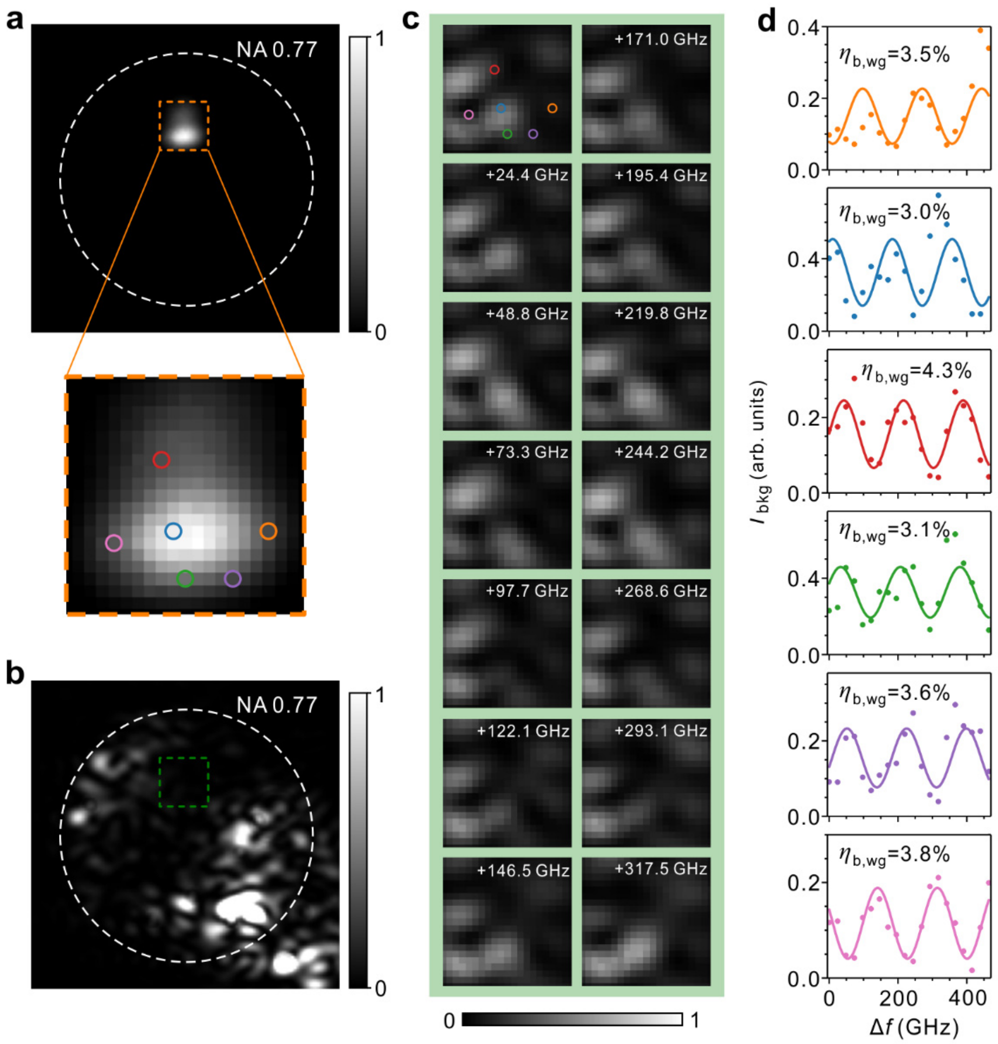

Supplementary Fig. 13 | Estimation of the waveguide-coupled laser background from GC2. a Fourier-plane image of the GC2-output 00ZPL emission of a single molecule under 0-1 excitation. The excitation laser is spectrally filtered. The colored circles in the zoom-in pattern denote the sampling positions. **b** Fourier-plane image of the laser background. Here the excitation is tuned 200 GHz away from the 00ZPL transition of the molecule and PH-R and polarization filtering have been applied. **c** Background intensity pattern within the PH-F (dashed rectangle in **b**) as the change of the laser frequency (presented at the upper-right corner of each pattern). Two columns correspond to two periods of pattern evolution. **d** Background intensity as a function of the laser detuning. Each color-coded trace corresponds to a position marked in the first graph of **c**.

Supplementary Note 7 – Determination of the excited-state lifetime

Lifetime τ_1 is extracted from the measured second-order cross-correlation function $g^{(2)}(\tau)$ shown in Fig. 4c of the main text. In the ideal case without background, the correlation function can be generally expressed as $g^{(2)}(\tau)_{\text{ideal}} = 1 - \frac{p+q}{2q} e^{-\frac{1}{2}(p-q)\tau} + \frac{p-q}{2q} e^{-\frac{1}{2}(p+q)\tau}$, with $p = \tau_1^{-1} + \tau_2^{-1}$, $q = \sqrt{(\tau_1^{-1} - \tau_2^{-1})^2 - 4S\tau_1^{-1}\tau_2^{-1}}$, i.e., dependent on the saturation parameter *S*, the coherence time τ_2, as well as the lifetime τ_1 [10]. With background counts (including the laser background and detector dark counts, which are uncorrelated), the correlation function is modified to $g^{(2)}(\tau) = 1 + \left(\frac{\text{SBR}_\text{d}}{\text{SBR}_\text{d} + 1}\right)^2 [g^{(2)}(\tau)_{\text{ideal}} - 1]$ [11], where SBR_d is the signal-to-background ratio with the APD dark count rate included in the background. The SBR_d averaged for the two channels is 45 for the case in Fig. 4c of the main text. The measured correlation function is fit with the lifetime τ_1 as the only free parameter since all the other relevant parameters are already known. The fitting yields $\tau_1 = 3.89 \pm 0.38$ ns. The orange line in Fig. 4c of the main text is the correlation function according to $g^{(2)}(\tau) = 1 + \left(\frac{\text{SBR}}{\text{SBR} + 1}\right)^2 [g^{(2)}(\tau)_{\text{ideal}} - 1]$, where the SBR averaged for the two channels has a value of 104 calculated for the pure background (with the APD dark count rate subtracted).

Supplementary Note 8 – Characterizations of RF saturation and coupling efficiency into the waveguide

a. RF saturation measurement

The RF-excitation spectra of the single molecule at varied laser excitation powers are measured and shown in Supplementary Fig. 14a. The peak intensities of the measured RF-excitation spectra are extracted as the detected RF intensities I_{det} and plotted in Supplementary Fig. 14b versus the excitation power (top horizontal axis). The power-dependent RF intensity I_{det} can be well fit with the theoretical saturation curve $I_{\text{det}} = I_{\infty}S/(1+S)$ (orange solid line in Supplementary Fig. 14b), where $S = P/P_{\text{sat}}$ is the saturation parameter with P_{sat} denoting the saturation excitation power level and I_{∞} is the saturated RF intensity. From the fitting, we obtain $P_{\text{sat}} = 14.96 \pm 0.67$ nW and $I_{\infty} = 42.90 \pm 1.08$ kcps. The saturation parameter is determined for any excitation power via $S = P/P_{\text{sat}}$.

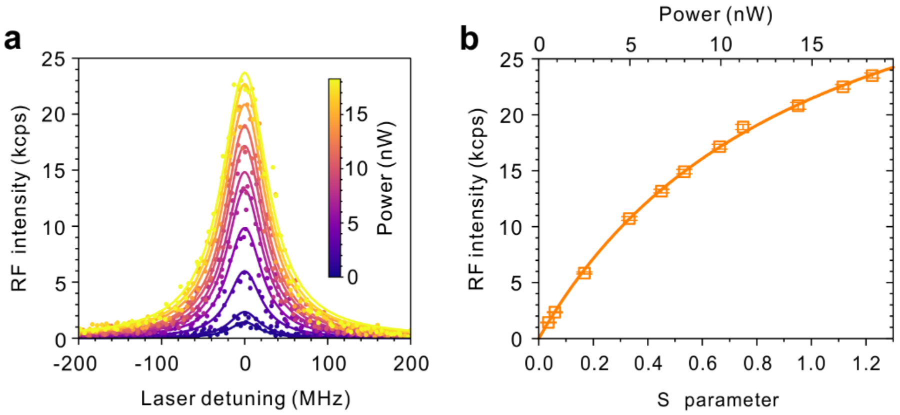

Supplementary Fig. 14 | RF saturation measurement. a RF-excitation spectra of the single molecule at varied laser excitation powers. **b** Peak intensity extracted from the measured RF-excitation spectra as a function of the saturation parameter S (bottom horizontal axis) or the excitation power P (top horizontal axis). The solid line is the theoretical fit $I_{\infty}S/(1+S)$.

b. Grating coupling efficiency measurement

The grating coupling efficiency is measured on a photonic circuit where two grating couplers with the same parameters are connected via the waveguide. A laser beam is launched onto one grating coupler and the output power from the other grating coupler is detected. The polarization of the laser beam matches the grating coupler. The incident angle is also optimized to maximize the output power P_{out} from the other grating coupler. The incident laser power P_{in} is obtained by measuring the reflected power for laser light impinging on the bare substrate

with the consideration of the reflection coefficient. In practice, the sample is simply moved laterally so that the laser beam is on the bare substrate. We assume that the coupling efficiencies of the input grating and output grating are the same, so that the coupling efficiency of the grating coupler can be obtained as $\eta_{\mathrm{GC}} = \sqrt{P_{\mathrm{out}}/P_{\mathrm{in}}}$, which is 8.6%.

c. RF coupling efficiency into the waveguide mode

The coupling efficiency of the molecule emission into waveguide mode (β factor) can be estimated by comparing the emitted photon count rate I_{em} with the detected photon count rate I_{det}[12]. The estimated 00ZPL photon emission rate can be expressed as

$$I_{\mathrm{em}} = \frac{\alpha\eta_{\mathrm{mol}}}{\tau_1}\frac{S}{1+S}, \tag{6}$$

where $\alpha = 0.33$ is the branching ratio to the 00ZPL for DBT in anthracene[13], the quantum efficiency of the molecule η_{mol} is taken as unity, $\tau_1 = 3.89$ ns is the excited-state lifetime (Supplementary Section 7). The detected photon count rate I_{det} can be expressed with I_{em} and β factor as

$$I_{\mathrm{det}} = \frac{1}{2}\beta\eta_{\mathrm{wg}}\eta_{\mathrm{GC}}\eta_{\mathrm{det}}I_{\mathrm{em}}, \tag{7}$$

where $\eta_{\mathrm{wg}} = 89\,\%$ is the numerically calculated transmittance of the photonic circuit considering only the insertion loss due to the termination of the AC nanosheet and the 2×2 MMI splitter, $\eta_{\mathrm{GC}} = 8.6\%$ is the measured coupling efficiency of the grating coupler, $\eta_{\mathrm{det}} = 16.5\%$ is the estimated optical detection efficiency taking into account both the transmittance of the optical elements and the quantum efficiency of the APD. The factor 1/2 is due to unidirectional detection. I_{det} has been shown in Supplementary Fig. 14b as a function of the saturation parameter *S* and is fitted as

$$I_{\mathrm{det}} = I_{\infty}\frac{S}{1+S}, \tag{8}$$

where the saturated intensity I_{∞} is determined to be 42.90 kcps. Substituting Supplementary Equations (6) and (8) into Supplementary Equation (7), β can be obtained as

$$\beta = \frac{2I_{\infty}\tau_1}{\alpha\eta_{\mathrm{mol}}\eta_{\mathrm{wg}}\eta_{\mathrm{GC}}\eta_{\mathrm{det}}} = 8\%.$$

Supplementary Note 9 – Molecules with unprecedented spectral stability

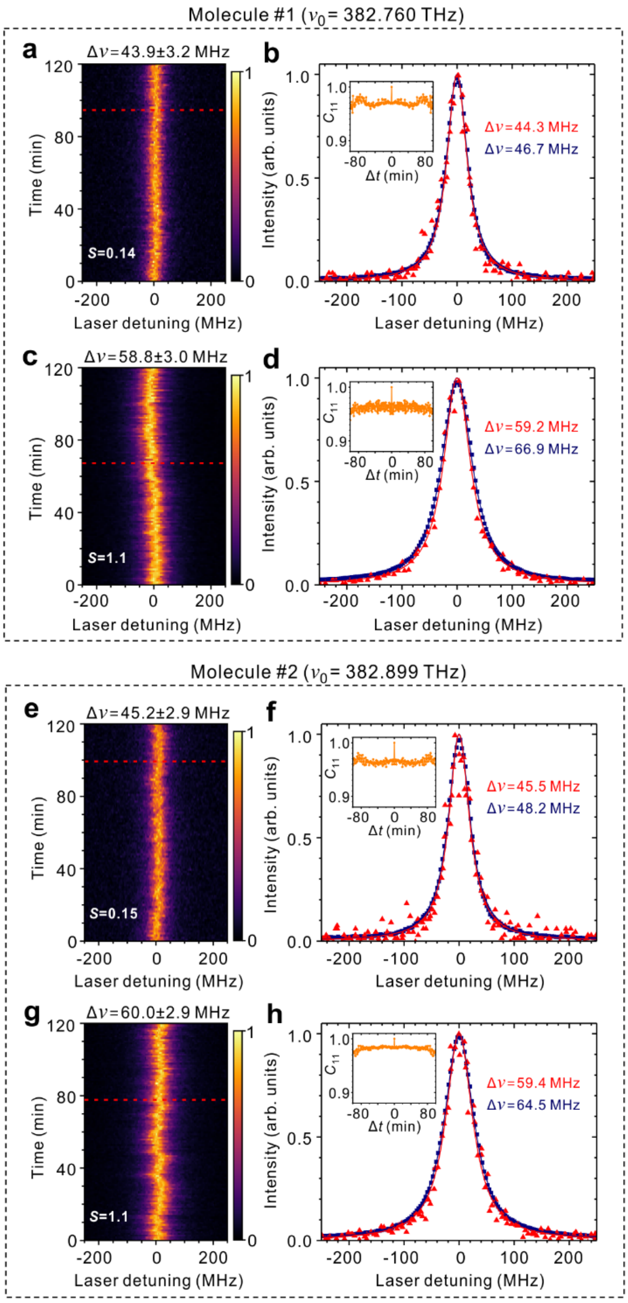

Supplementary Fig. 15 | Spectral stability under resonant excitation. a-d Spectral stability of molecule #1. **a** Fluorescence-excitation spectra (S = 0.14) recorded for two hours with the excitation laser scanned across the 00ZPL at a speed of 200 MHz/s. **b** Superposition of all recorded fluorescence-excitation spectra (blue) and a typical single-scan fluorescence-excitation spectrum (red) recorded at the time marked by the red dashed line in **a**. Inset: normalized spectral autocorrelation as a function of time delay. **c**, **d** Same as **a** and **b**, respectively, but S = 1.1 (this value of S leads to a power broadening of the linewidth by 45%). **e-h** Spectral stability of molecule #2. **e** Fluorescence-excitation spectra (S = 0.15) recorded for two hours with the excitation laser scanned across the 00ZPL at a speed of 200 MHz/s. **f** Superposition of all recorded fluorescence-excitation spectra (blue) and a typical single-scan fluorescence-excitation spectrum (red) recorded at the time marked by the red dashed line in **e**. Inset: normalized spectral autocorrelation as a function of time delay. **g**, **h** Same as **e** and **f**, respectively, but S = 1.1 (this value of S leads to a power broadening of the linewidth by 45%).

Supplementary Fig. 15 displays the spectral stability under resonant excitation for two more molecules (Molecule #1, Molecule #2), both showing only slight inhomogeneous broadening due to spectral diffusion within two hours.

Supplementary References

1. Wei, S., Ren, P., He, Y., Zhang, P. & Chen, X.-W. Single-Molecule-Doped Crystalline Nanosheets for Delicate Photophysics Studies and Directional Single-Photon-Emitting Devices. *Physical Review Applied* **13**, 064023 (2020).
2. Nakada, I. The Optical Properties of Anthracene Single Crystals. *Journal of the Physical Society of Japan* **17**, 113-118 (1962).
3. Matsui, A. & Ishii, Y. Optical Properties of Anthracene Single Crystals. *Journal of the Physical Society of Japan* **23**, 581-590 (1967).
4. Cummins, P. G. & Dunmur, D. A. The electric permittivity of crystalline anthracene. *Journal of Physics D: Applied Physics* **7**, 451-454 (1974).
5. Julian, M. M. & Bloss, F. D. Optical measurements of anthracene. *Acta Crystallographica Section A* **38**, 167-169 (1982).
6. Chen, Y. T., Nielsen, T. R., Gregersen, N., Lodahl, P. & Mork, J. Finite-element modeling of spontaneous emission of a quantum emitter at nanoscale proximity to plasmonic waveguides. *Physical Review B* **81**, 125431 (2010).
7. Quan, Q. M., Bulu, I. & Loncar, M. Broadband waveguide QED system on a chip. *Physical Review A* **80**, 011810(R) (2009).
8. Zhang, P., Ren, P. L. & Chen, X. W. On the emission pattern of nanoscopic emitters in planar anisotropic matrix and nanoantenna structures. *Nanoscale* **11**, 11195-11201 (2019).
9. He, Y., Lin, S. P., Robert, H. M. L., Li, H., Zhang, P., Piliarik, M. & Chen, X. W. Multiscale modeling and analysis for high-fidelity interferometric scattering microscopy. *Journal of Physics D-Applied Physics* **54**, 274002 (2021).
10. Grandi, S., Major, K. D., Polisseni, C., Boissier, S., Clark, A. S. & Hinds, E. A. Quantum dynamics of a driven two-level molecule with variable dephasing. *Physical Review A* **94**, 063839 (2016).
11. Lettow, R., Rezus, Y. L. A., Renn, A., Zumofen, G., Ikonen, E., Götzinger, S. & Sandoghdar, V. Quantum Interference of Tunably Indistinguishable Photons from Remote Organic Molecules. *Physical Review Letters* **104**, 123605 (2010).
12. Lombardi, P., Ovvyan, A. P., Pazzagli, S., Mazzamuto, G., Kewes, G., Neitzke, O., Gruhler, N., Benson, O., Pernice, W. H. P., Cataliotti, F. S. & Toninelli, C. Photostable Molecules on Chip: Integrated Sources of Nonclassical Light. *ACS Photonics* **5**, 126-132 (2018).
13. Trebbia, J. B., Ruf, H., Tamarat, P. & Lounis, B. Efficient generation of near infra-red single photons from the zero-phonon line of a single molecule. *Optics Express* **17**, 23986-23991 (2009).
